# Biocompatible nanoscale contrast agents for MRI and CT scans: current progress and clinical potential

**DOI:** 10.3762/bjnano.17.104

**Published:** 2026-11-02

**Authors:** Mohammad Mehdi Gravandi, Pershia Davoodi Karsalari, Ali Hajihashemi, Shokouh Sadeghizadeh, Mohammad Rasoul Golabchi, Ghazal Mohammadbeigi, Mahsa Geravandi, Niayesh Mohebbi

**Affiliations:** 1 Pharmaceutical Sciences Research Center, Health Institute, Kermanshah University of Medical Sciences, Kermanshah, Iran; 2 Pediatric Urology and Regenerative Medicine Research Center, Gene, Cell, and Tissue Research Institute, Children’s Medical Center, Tehran, Iran; 3 Department of Radiology, Isfahan University of Medical Sciences, Isfahan, Iran; 4 Division of Nanobiotechnology, Department of Life Science Engineering, Faculty of Interdisciplinary Science and Technology, University of Tehran, Tehran, Iran; 5 Department of Clinical Pharmacy, Faculty of Pharmacy, Tehran University of Medical Sciences, Tehran, Iran

**Keywords:** biocompatible nanoscale contrast agents, clinical translation, computed tomography (CT) scan, diagnostic accuracy, magnetic resonance imaging (MRI), nanoparticle pharmacokinetics, non-invasive imaging

## Abstract

Conventional contrast agents for magnetic resonance imaging (MRI) and computed tomography (CT) have long served as the backbone of diagnostic imaging. Yet, their well-recognized limitations, including nephrogenic systemic fibrosis and progressive gadolinium deposition with Gd-based chelates, contrast-induced nephropathy with iodinated agents, and serious hypersensitivity reactions with approved iron oxide formulations, continue to drive the search for safer alternatives. Nanoparticle-based contrast agents (NCAs) have emerged as a compelling next generation of imaging tools, offering a degree of rational design simply not achievable with small-molecule agents. By independently tuning core composition, hydrodynamic size, surface chemistry, and morphology, NCAs can be engineered to enhance relaxivity or X-ray attenuation, extend vascular residence time, direct clearance toward renal or hepatobiliary pathways, and carry targeting ligands for site-specific delivery. This review covers the principal NCA classes, iron oxide nanoparticles, gadolinium- and manganese-based nanomaterials, gold and copper nanostructures, carbon dots, lanthanide-doped upconversion nanocrystals, and quantum dots, examining their physicochemical design, pharmacokinetics, biocompatibility, and diagnostic performance across oncological and vascular applications. A recurring theme is the central role of size: Particles below the ≈6 nm glomerular filtration threshold undergo renal excretion and exhibit lower bioaccumulation risk, while larger formulations follow hepatobiliary routes with implications for long-term tissue retention. Surface coating, protein corona formation, and mononuclear phagocyte system recognition are discussed as equally decisive determinants of in vivo behavior, often outweighing core composition in their influence on clearance and tolerability. Across material classes, preclinical evidence consistently shows favorable safety profiles for well-engineered NCAs, with minimal hemolysis, low immunogenicity, and absence of histological organ injury at imaging-relevant doses, a meaningful contrast to the adverse effect profiles of conventional agents. On the efficacy side, targeted NCA formulations have demonstrated promising results across multiple tumor types, including breast, brain, liver, lung, and renal cancers, with receptor-specific designs enabling molecular-level lesion characterization that conventional agents cannot provide. Multimodal and theranostic constructs, combining MRI or CT contrast with concurrent therapeutic function, further illustrate the flexibility that nanoparticulate platforms offer. Blood–brain barrier penetration for neurooncological applications and the emerging potential of magnetic particle imaging as a future translational direction for platforms based on superparamagnetic iron oxide nanoparticles are also discussed. The translational gap between preclinical promise and clinical reality remains the field’s most honest challenge, rooted in the scarcity of long-term human safety data, the lack of standardized preclinical evaluation frameworks, and the demanding requirements of good-manufacturing-practice production at scale. Addressing these gaps, through unified safety pipelines, harmonized reporting standards, and early-phase trials that connect imaging performance to clinical outcomes, is what will ultimately determine whether NCAs fulfill their considerable potential in diagnostic medicine.

## Review

### 1 Introduction

Contrast agents are essential for enhancing diagnostic capabilities of magnetic resonance imaging (MRI) and computed tomography (CT) scans by improving tissue differentiation and structural visualization [1–2]. Nanoparticle-based CAs (NCAs) have emerged to address the principal limitation of conventional agents, that is, the short blood half-life of small-molecule agents of typically 10–20 min for iodinated CT agents and less than 30 min for extracellular gadolinium-based CAs (GBCAs) before redistribution into the interstitium, which restricts the available imaging window and prevents meaningful tumor accumulation [3].

In contrast, surface-engineered NCAs exploit size-dependent renal filtration thresholds and steric stabilization to extend vascular residence to several hours, enabling delayed and steady-state imaging protocols inaccessible with conventional agents [3–4]. Their nanoparticulate architecture also confers tunable physicochemical properties, including core composition, hydrodynamic diameter, surface charge, and coating chemistry, which can be independently optimized to modulate relaxivity or X-ray attenuation, control biodistribution, and attach targeting ligands, affording a degree of rational design not achievable with small-molecule chelates [4]. However, their biocompatibility, biodegradability, and clearance mechanisms remain critical concerns requiring thorough evaluation before clinical translation [5–6]. While numerous preclinical and early clinical studies have investigated NCAs, a comprehensive synthesis of their safety, pharmacokinetics, and comparative effectiveness is lacking [7–8]. The following sections explain the physical basis for the advantages of NCAs.

MRI generates images by exploiting the nuclear magnetic resonance properties of hydrogen protons abundant in biological tissues. When placed in a strong external magnetic field, protons align with the field and precess at a characteristic Larmor frequency proportional to the field strength. A radiofrequency (RF) pulse applied at this frequency tips the net magnetization vector away from equilibrium; upon cessation of the pulse, protons return to their resting state through two simultaneous but independent relaxation processes. Longitudinal relaxation (*T*_1_) describes the time constant for recovery of magnetization along the axis of the external field through energy exchange with the surrounding molecular lattice, while transverse relaxation (*T*_2_) reflects the decay of magnetization in the perpendicular plane due to spin–spin interactions and local magnetic field inhomogeneities. The spatial variation of *T*_1_ and *T*_2_ across tissues, governed by their molecular composition, water content, and macromolecular structure, constitutes the intrinsic source of soft tissue contrast in MRI. Contrast agents amplify these differences by altering the local magnetic environment of adjacent water protons. Paramagnetic agents such as Gd^3+^ chelates possess unpaired electrons whose strong magnetic moment accelerates both *T*_1_ and *T*_2_ relaxation, predominantly shortening *T*_1_ and producing signal enhancement on *T*_1_-weighted sequences, while superparamagnetic iron oxide nanoparticles (SPIONs) generate large local field inhomogeneities that drastically shorten *T*_2_ and *T*_2_*, producing signal loss on *T*_2_-weighted sequences. The efficiency of this effect is quantified as relaxivity (*r*_1_ or *r*_2_, in mM^−1^·s^−1^), which directly governs how much contrast enhancement a given concentration of agent produces at a given field strength and, therefore, how the physicochemical properties such as size, surface chemistry, and core composition of the NCA translate into diagnostic image quality [9–10].

CT operates on fundamentally different physical principles, using X-ray radiation rather than magnetic fields. A rotating X-ray source emits a polychromatic beam that traverses the patient at multiple angles; detectors on the opposite side measure the transmitted intensity at each projection. The degree to which tissues attenuate the beam, their linear attenuation coefficient, depends on their physical density and effective atomic number, and varies with photon energy. These attenuation measurements are reconstructed computationally into cross-sectional images in which each voxel is assigned a CT number expressed in Hounsfield units (HU), calibrated so that water equals 0 HU and air equals −1000 HU. Bone, with high density and calcium content, appears bright (400–1000 HU), while soft tissues cluster in a narrow range (20–80 HU) that is difficult to differentiate without contrast enhancement. Contrast agents for CT function by locally increasing X-ray attenuation; iodine and other high-atomic-number elements are effective because the photoelectric attenuation cross section scales approximately with the cube of the atomic number, meaning that elements such as iodine (*Z* = 53), gold (*Z* = 79), or bismuth (*Z* = 83) attenuate X-rays far more strongly than soft tissue at diagnostic energies. Conventional small-molecule iodinated agents achieve this by rapidly spreading into the extravascular space, providing a short vascular imaging window. NCAs exploit the same attenuation principle but with nanoparticle architectures that extend the vascular phase, enable passive or active tumor accumulation, and in the case of photon-counting CT, allow for K-edge imaging at element-specific energies for spectral tissue decomposition [2,11]. Together, these physical principles define the design requirements that NCAs must satisfy to outperform conventional agents in each modality. In practice, these gains are quantitatively substantial: Selected Gd-based NCAs have demonstrated *r*_1_ values two- to threefold higher than clinical gadolinium diethylenetriamine pentaacetic acid (Gd-DTPA), approximately 3–5 mM^−1^·s^−1^ at 1.5 T), Mn-based nanoparticulate systems have achieved *r*_1_ values exceeding 20 mM^−1^·s^−1^, and high-atomic-number NCAs such as gold and bismuth nanoparticles yield markedly greater X-ray attenuation per unit mass than iodine at diagnostic energies, collectively enabling superior contrast at equivalent or reduced doses [12–13]. This review consolidates current evidence, highlighting physiological interactions, clearance pathways, and potential clinical applications, providing insights to support the safe and effective translation of NCAs into practice.

### 2 Biocompatible NCAs

An important advancement in medical imaging is the development of biocompatible NCAs for MRI and CT scans. CT provides high-resolution imaging with deep tissue penetration and cost-effectiveness, whereas MRI offers non-invasive imaging with superior soft-tissue contrast and no ionizing radiation. However, the intrinsic limitations of these modalities, such as lower soft-tissue sensitivity in CT or limited bone visualization in MRI, often necessitate the use of contrast agents to enhance diagnostic accuracy [14–16]. Combining CT and MRI in multimodal imaging further improves diagnostic efficiency by compensating for the limitations of each modality. Conventional contrast agents, such as small-molecule iodinated compounds for CT or gadolinium-based complexes for MRI, are typically single-modality agents associated with lower sensitivity, potential allergic reactions, and renal toxicity [16].

Currently, the most common MRI contrast agents used in clinical practice are gadolinium (Gd)-based compounds, which produce positive contrast through complexes formed between the central Gd^3+^ ion and a chelating ligand. Despite their diagnostic value, these agents may cause serious side effects such as nephrogenic systemic fibrosis (NSF) and long-term brain tissue deposition due to the release of free gadolinium ions [17–19]. These challenges have accelerated the development of biocompatible NCAs that prioritize improved safety, reduced systematic toxicity, and support for dual-modality imaging, thereby expanding diagnostic applications [3]. Iodinated contrast agents, the conventional choice for CT, present a distinct biocompatibility profile. Their principal safety concerns are hypersensitivity reactions, ranging from mild urticaria to severe anaphylaxis, and contrast-induced nephropathy, most commonly defined as a substantial rise in serum creatinine following administration. These risks are influenced by agent osmolarity, with high-osmolar ionic agents associated with a higher incidence of adverse reactions than newer low- or iso-osmolar nonionic formulations, and they are compounded in patients with pre-existing renal impairment or diabetes. As with gadolinium-based MRI agents, these limitations have motivated the development of nanoparticle-based CT contrast platforms designed to reduce required iodine doses, improve renal clearance profiles, and lower the incidence of hypersensitivity reactions [20–21]. Contrast agents enhance the visibility of internal structures, which improves the diagnostic accuracy of MRI and CT scans. This improved clarity has further expanded their clinical applications in medical imaging [22]. Biocompatibility, in the context of NCAs, refers to the ability of a material to interact safely with the host and be effectively cleared from the body after fulfilling its diagnostic role [23].

Biocompatibility of NCAs is governed largely by their interaction with the biological environment immediately following administration. Upon entering the bloodstream, nanoparticles rapidly adsorb a layer of plasma proteins known as the protein corona, which redefines the particles’ effective biological identity and strongly influences circulation time, cellular uptake, and immune recognition. Opsonins within this corona, including complement proteins, immunoglobulins, and fibrinogen, can be recognized by phagocytic cells of the mononuclear phagocyte system, triggering sequestration in the liver and spleen, complement activation, and inflammatory responses. Conversely, dysopsonins such as albumin can reduce immune recognition and prolong circulation. Surface engineering strategies, including surface conjugation with polyethylene glycol (PEGylation) and zwitterionic or biomimetic coatings, are commonly employed to modulate corona composition and minimize unwanted immune activation, directly improving the biocompatibility profile of NCAs [24–25]. Particle size distribution is a critical determinant of NCA biocompatibility, primarily through its effect on renal clearance. The glomerular filtration barrier imposes a size-dependent threshold: Nanoparticles with a hydrodynamic diameter below 5.5–6.0 nm are efficiently filtered and excreted in urine, while particles above roughly 8 nm are largely excluded from renal filtration and instead accumulate in organs of the reticuloendothelial system, particularly the liver and spleen, raising the risk of long-term retention and toxicity. This renal cutoff has become a key design target for biocompatible NCAs, motivating efforts to keep nanoparticle cores or fully assembled constructs below this threshold wherever diagnostic performance allows. At the same time, very small particles may suffer from shortened circulation half-lives that limit target accumulation, illustrating a fundamental trade-off between rapid, safe clearance and sufficient residence time for effective imaging [26–27].

NCAs can also incorporate multiple paramagnetic ions or molecular markers to improve contrast and specificity, while their pharmacokinetics, including circulation half-life and target-site retention, can be tuned by adjusting particle size, shape, or surface chemistry [1,28]. The morphology of nanoparticles is an independently important design parameter that influences imaging performance, biodistribution, cellular uptake, and clearance through several distinct mechanisms. Common NCA morphologies include nanospheres, nanocubes, nanorods, nanoplates, and hollow or core–shell structures, each conferring distinct physicochemical behavior [29]. With respect to MRI contrast, anisotropic shapes such as nanorods and nanocubes generate stronger local magnetic field inhomogeneities than spheres of equivalent volume, owing to their higher surface-area-to-volume ratios and shape-dependent magnetic anisotropy, which translates into higher *r*_2_ relaxivity and more pronounced *T*_2_ contrast [30]. For CT, rod- and plate-shaped high-atomic-number nanoparticles present a larger projected cross section per unit mass along certain orientations, enhancing X-ray attenuation efficiency relative to spheres at equivalent elemental loading [29]. With respect to biodistribution and clearance, shape governs the dynamics of migration toward vessel walls in blood flow: Rod-shaped particles tumble and drift laterally more efficiently than spheres under shear flow conditions, increasing their probability of contact with endothelial surfaces and thereby enhancing passive vascular wall accumulation. Elongated particles also interact differently with the mononuclear phagocyte system. Macrophage uptake is strongly shape-dependent, with high-aspect-ratio rods being internalized more slowly than spheres of comparable size due to the geometry of membrane wrapping required for phagocytosis, potentially extending the circulation time [31]. Nanocubes, by contrast, exhibit flat facets that promote closer contact with cell membranes, facilitating receptor–ligand interactions and improving active targeting efficiency compared to spheres [30]. These morphology-dependent differences in relaxivity, circulation, and cellular uptake underscore that shape is not a secondary parameter but a primary design handle that should be co-optimized with size and surface chemistry in rational NCA development.

Several types of nanomaterials have been explored as contrast agents, including gold-based composites such as Fe_3_O_4_@Au, MnO_2_ nanosheets, silica nanoparticles, and carbon-based materials like graphene, carbon nanospheres, and carbon quantum dots [32]. These systems have shown promise for dual-modality CT/MRI applications [16]. Many NCAs are designed with modified inorganic cores and multifunctional surface features that improve circulation, promote tissue accumulation, and enhance tumor visualization [14]. Key limitations include restricted biodegradability, inefficient renal clearance of particles above the 6–8 nm glomerular filtration threshold, and accumulation within reticuloendothelial organs such as the liver and spleen, which may contribute to toxicity [28]. Standard assessments of NCA safety typically involve cytotoxicity testing, hemolysis assays, serum biochemistry, and histological evaluation [32]. Continued research is needed to clarify their biodegradation profiles, clearance mechanisms, and long-term safety to support successful clinical translation [33].

### 3 Nanoparticle-based contrast agents in MRI and CT

Building on their potential advantages, NCAs have been developed to enhance diagnostic precision while minimizing toxicity, particularly in MRI and CT applications [34–35].

#### 3.1 Overview

NCAs have emerged as next-generation alternatives to conventional low-molecular-weight (LMW) Gd^3+^ chelates used in MRI, offering tunable physicochemical properties such as core composition, size, and surface coating to enhance relaxivity and extend circulation time. NCAs provide opportunities for dual-mode MRI (*T*_1_/*T*_2_) or MRI/CT integration, facilitating high-resolution and multimodal diagnostics. To address limitations of conventional contrast agents, new nanoscale agents are being developed to enhance vascular retention, improve relaxivity indices, and reduce toxicity, while also being explored for targeted therapy and drug delivery [1,6,12].

#### 3.2 Classification by core composition

NCAs are commonly classified according to their core materials, which determine their magnetic behavior and relaxometric performance.

**3.2.1 Iron oxide nanoparticles.** Superparamagnetic iron oxide nanoparticles, primarily composed of magnetite (Fe_3_O_4_) and maghemite (γ-Fe_2_O_3_), have been widely investigated for biomedical applications. These nanoparticles possess high magnetic moments, large specific surface areas, and minimal renal accumulation, supporting their use in magnetic separation, biosensing, hyperthermia- and photothermia-based therapy, targeted drug or gene delivery, and as MRI contrast agents. Owing to their favorable biocompatibility and intrinsic magnetic properties, iron oxide nanoparticles (IONPs) are considered among the most promising inorganic platforms for clinical translation. Because iron is naturally present in the human body, IONPs typically exhibit higher biocompatibility than Gd^3+^-based contrast agents [17]. The hydrodynamic diameter of SPIONs varies widely, from a few nanometers to more than 100 nm [36]. It is important to note that nanoparticle size is not a single-valued property: The hydrodynamic diameter, measured by dynamic light scattering, includes the inorganic core, surface coating, and hydration shell; the physical diameter, determined by statistical analysis of transmission electron microscopy images, reflects the inorganic core alone; and the magnetic diameter, derived from fitting magnetization curves, captures only the magnetically responsive core volume, which may be smaller than the physical core due to surface spin disorder. Each metric conveys distinct information, and reporting size distribution, rather than a single mean value, is essential given the polydispersity inherent in most SPION preparations [37]. For example, Ferumoxytol (Feraheme), with a hydrodynamic diameter of ≈50 nm, functions as a *T*_2_-weighted contrast agent. In contrast, ultrasmall SPIONs (smaller than 5 nm) show reduced magnetic moments and can therefore produce *T*_1_-weighted enhancement. Both Fe_3_O_4_ and γ-Fe_2_O_3_ have been explored for in vivo magnetic targeting and drug delivery applications [38–39].

Although γ-Fe_2_O_3_ is chemically more stable than magnetite, Fe_3_O_4_ remains the preferred phase for contrast agent applications owing to its higher bulk saturation magnetization (≈92 emu/g versus ≈74 emu/g for γ-Fe_2_O_3_), which translates directly into superior MRI relaxivity and signal enhancement; this is reflected in the composition of both Feridex^®^ and Resovist^®^, whose iron oxide cores are predominantly magnetite [36]. The susceptibility of bare Fe_3_O_4_ to oxidative degradation to Fe_2_O_3_ is effectively mitigated through surface coating. Polymeric shells including dextran, carboxydextran, polyethylene glycol (PEG), and silica physically shield the iron oxide core from dissolved oxygen and biological oxidants, prevent aggregation, and preserve colloidal stability under physiological conditions, thereby maintaining the magnetic performance of the magnetite phase throughout the particle’s in vivo lifetime [40–41].

Magnetic targeting can be further enhanced by clustering multiple IONPs within a single carrier rather than relying on individual nanoparticles alone. For instance, hybrid polysaccharide microgels loaded with of the order of 10^5^ magnetite nanoparticles per particle have been shown to generate a strong collective magnetic macromoment (≈10^−11^ emu), substantially improving magnetic targeting efficiency both in vitro and in vivo. Such multiparticle assemblies illustrate a broader strategy for translating IONP magnetic properties into more effective targeted delivery, with corresponding implications for biocompatibility, since clustering and carrier composition influence biodistribution, clearance, and immune recognition relative to individual nanoparticles [42]. Magnetic nanocrystals markedly amplify MRI signal modulation and can outperform traditional small-molecule agents [43]. Broadly, contrast agents are divided into *T*_1_-positive agents (paramagnetic), which produce bright contrast, and *T*_2_-negative agents (superparamagnetic), which produce signal darkening [9–10]. Among *T*_2_ agents, SPIONs remain the most extensively studied [44–45]. However, their clinical utility is limited by susceptibility-induced signal-void artifacts and magnetic field distortions, which can mimic hemorrhage, calcification, or metal deposition on *T*_2_-weighted or gradient-echo MRI [10,45–46]. These susceptibility effects may also degrade background anatomical detail through local field inhomogeneities, commonly described as a “blooming effect” [40]. In contrast, *T*_1_-weighted contrast agents generate positive enhancement, aiding clearer tissue delineation and high-resolution anatomical imaging [39].

**3.2.2 Lanthanide-doped nanocrystals.** Lanthanide-doped upconversion nanocrystals (UCNCs) are widely applied in lasers, display technologies, data storage, sensing, and biomedical imaging. Their unique optical properties, deep-tissue penetration, stable and intense upconversion luminescence, and strong resistance to photobleaching, enable high-sensitivity imaging with minimal background fluorescence. UCNCs incorporating Gd^3+^ exhibit paramagnetic behavior at physiological temperatures, altering water proton relaxation times and providing MRI contrast. Moreover, lanthanide has a high atomic number, generating strong X-ray attenuation suitable for CT imaging. Collectively, Ln^3+^-doped UCNCs offer versatile capabilities for upconversion luminescence imaging (UCL), CT, and MRI applications [47].

The biocompatibility of lanthanide-doped nanocrystals is strongly influenced by the choice of the host matrix, which governs aqueous stability, ion-release behavior, and clearance pathways. Table 1 summarizes the principal host matrix classes and their associated biocompatibility-relevant characteristics. Oxide hosts (e.g., Y_2_O_3_ and Gd_2_O_3_) offer high chemical and thermal stability and low aqueous solubility, which limits ion leaching, though their relatively high phonon energy reduces upconversion efficiency compared with fluoride hosts. Fluoride hosts, particularly hexagonal NaLnF_4_ systems (e.g., NaYF_4_ and NaGdF_4_), are favored for their low phonon energy and superior upconversion efficiency, but are more susceptible to aqueous dissolution, releasing fluoride and lanthanide ions that have been linked to cytotoxicity; this dissolution behavior is strongly dependent on the crystal phase, with the metastable cubic α-phase generally more prone to dissolution than the thermodynamically stable hexagonal β-phase, although surface coating remains a major confounding factor in phase-dependent comparisons. Oxysulfide hosts (e.g., Gd_2_O_2_S) are characterized by very low aqueous solubility and have demonstrated high tolerability at substantial intravenous doses in rodent models, with clearance occurring predominantly through slow hepatobiliary pathways rather than renal excretion, raising considerations about longer-term tissue retention. Collectively, these matrix- and phase-dependent differences underscore that biocompatibility cannot be generalized across all Ln^3+^-doped nanocrystals and must instead be evaluated per host system [48–50].

**Table 1:** Host matrix effects on the biocompatibility of lanthanide-doped nanocrystals.

Host matrix class	Representative materials	Biocompatibility-relevant properties

oxides [48]	Y_2_O_3_, Gd_2_O_3_	high chemical/thermal stability; low aqueous solubility limits ion leaching; higher phonon energy lowers UC efficiency
fluorides (NaLnF_4_ and LnF_3_) [49]	NaYF_4_, NaGdF_4_, YF_3_	low phonon energy → high UC efficiency; susceptible to aqueous dissolution releasing F^−^/Ln^3+^; cytotoxicity linked to dissolution extent
oxysulfides [50]	Gd_2_O_2_S	very low aqueous solubility; high in vivo tolerability at substantial doses; slow hepatobiliary clearance (limited renal excretion)
crystal phase (fluorides) [49]	cubic α-NaYF_4_ vs hexagonal β-NaYF_4_	α-phase metastable, more prone to dissolution; β-phase more stable, generally improved biocompatibility; surface coating is a confounding factor

Preclinical safety data for UCNCs remain comparatively limited, but recent work has begun to establish their in vivo innocuity over clinically relevant timeframes: poly(ethylene oxide)–poly(acrylic acid)-coated (PEO-PAA-coated) NaYF_4_:Yb,Tm UCNCs injected into the myocardium or pericardial space were retained in cardiac tissue for up to 15 days without altering heart rate, heart rate variability, or left ventricular ejection fraction, and with minimal to no cardiac tissue necrosis when administered into the pericardial space [51]. A comparison of pharmacokinetic and biocompatibility parameters across NCA material classes, including this lanthanide-based example, is provided below in Table 2.

**3.2.3 Manganese-based nanoparticles.** Mn-based nanoparticles serve as effective *T*_1_-weighted MRI contrast agents, with lower toxicity than Gd^3+^-based complexes. Manganese naturally participates in cellular metabolism and is efficiently absorbed and excreted, contributing to its favorable safety profile. Due the physicochemical similarities to Gd-based contrast agents, Mn-based nanomaterials provide strong positive contrast for *T*_1_ imaging. In particular, MnO_2_ nanocomplexes decompose in the tumor microenvironment, releasing Mn^2+^, which is subsequently cleared by the kidneys. Beyond MnO_2_, several other manganese oxidation states and structures have been explored as MRI contrast platforms. MnO (Mn^2+^) nanoparticles represent the most extensively studied class, alongside Mn_3_O_4_ (mixed Mn^2+^/Mn^3+^) and Mn_2_O_3_ (Mn^3+^), which differ in magnetic behavior and relaxivity. Manganese ferrite (MnFe_2_O_4_) nanoparticles constitute a structurally distinct class in which Mn^2+^ and Fe^3+^ cations occupy a spinel lattice, conferring strong superparamagnetic behavior that supports their use primarily as *T*_2_-weighted contrast agents, in addition to applications in magnetic hyperthermia [52–53]. The tumor-selective decomposition of MnO_2_ nanocomplexes arises from two characteristic features of the tumor microenvironment relative to healthy tissue, namely a mildly acidic extracellular pH (typically 6.5–6.8, versus ≈7.4 in normal tissue) resulting from elevated glycolytic activity, and markedly elevated intracellular glutathione (GSH) concentrations associated with the redox imbalance of tumor cells. Both H^+^ and GSH reduce Mn^4+^ to Mn^2+^, driving dissolution of the MnO_2_ lattice; the same reaction is frequently coupled to decomposition of tumor-associated hydrogen peroxide into oxygen, which can additionally help relieve tumor hypoxia. This tumor-responsive behavior provides enhanced contrast while maintaining low systemic toxicity [54–56].

**3.2.4 Carbon dots.** Carbon dots (C-dots), a rapidly expanding class of NCAs, are widely used in fluorescence imaging and drug delivery due to their excellent biocompatibility, high aqueous solubility, tunable surface functionalization, and stable photoluminescence. Their ultrasmall size enables efficient renal clearance. C-dots below 5–6 nm in hydrodynamic diameter undergo glomerular filtration and begin accumulating in the bladder within minutes of intravenous injection in murine models, with urinary excretion largely completed within 24 h [57]. This clearance profile is broadly comparable in timescale to that of the clinical gold-standard Gd-DTPA, which is eliminated renally with a plasma half-life of approximately 1.5 h in patients with normal renal function, though the mechanistic basis differs: Gd-DTPA is a small-molecule chelate cleared entirely by glomerular filtration, whereas C-dot clearance is size-gated at the glomerular filtration threshold and strongly depends on surface charge and coating [57–58]. C-dots below 5 nm have additionally demonstrated minimal cytotoxicity in preclinical studies [57]. However, their ultrasmall dimensions typically result in reduced circulation time, which may limit imaging duration and target accumulation [55,59]. Beyond fluorescence, C-dots have been established as viable NCAs for both MRI and CT through heteroatom and metal-ion doping. For MRI, incorporation of Gd^3+^, Mn^2+^, or Fe^3+^ ions into the carbon matrix generates paramagnetic or superparamagnetic properties that enhance proton relaxation. Gd/Ru-doped C-dots have demonstrated *r*_1_ values of 6.38 mM^−1^·s^−1^ at 3.0 T, comparable to Gd-DTPA, while Gd/Yb co-doped C-dots achieved *r*_1_ = 6.65 mM^−1^·s^−1^ alongside CT attenuation of 45.43 HU·L·g^−1^, exceeding that of the clinical CT agent iobitridol (31.83 HU·L·g^−1^). For CT, heavy atom doping with iodine, hafnium, or lanthanides (ytterbium, gadolinium) exploits the high atomic number of these elements to generate X-ray attenuation, enabling C-dots to function as dual MRI/CT or triple MRI/CT/fluorescence agents. A key limitation of metal-doped C-dots for clinical translation relates to post-synthesis functionalization. The surface carboxyl, hydroxy, and amine groups that govern colloidal stability and biocompatibility are the same groups used for metal chelation during doping and for attachment of targeting ligands. When these groups are occupied by dopant metals, the available chemistry for further surface modification is reduced, constraining the flexibility to independently tune targeting, biocompatibility, and imaging performance. This design trade-off is compounded by broader translational challenges. Synthetic reproducibility and inadequate purification remain major obstacles, since unbound metal ions or fluorescent molecular by-products generated during synthesis are frequently mistaken for the intrinsic properties of the carbon dots themselves, underscoring the need for rigorous purification and characterization protocols before clinical translation can advance [60–62].

**3.2.5 Quantum dots.** A related class of fluorescent semiconductor nanoparticles, quantum dots (QDs), warrants mention alongside carbon dots given their structural and functional similarities in bioimaging contexts. Unlike carbon dots, which are made from organic carbon precursors, QDs are inorganic semiconductor nanocrystals (e.g., CdSe, CdTe, InP, or ZnS) whose optical properties arise primarily from quantum confinement effects rather than surface chemistry. For CT imaging specifically, QDs incorporating high-atomic-number elements have been explored as alternatives to iodinated agents, with recent reviews demonstrating their potential for both monomodal and bimodal CT applications. However, the biocompatibility profile of QDs, particularly heavy-metal-based formulations containing cadmium or tellurium, remains a significant translational concern. Core toxicity from metal ion leaching, incomplete renal clearance of larger QDs, and dose-dependent cytotoxicity have all been documented in preclinical models. Heavy-metal-free alternatives (InP QDs and carbon-based QDs) have been developed to address these concerns, though their imaging performance in CT and MRI generally remains inferior to other formulations. A comprehensive discussion of QDs as NCAs falls beyond the primary scope of this review, but their ongoing development as CT and multimodal contrast platforms is noted here as a complementary direction to the carbon-based systems described above [63].

**3.2.6 Liposomes and magnetoliposomes.** Liposomes are spherical self-assembled vesicles composed of one or more phospholipid bilayers enclosing an aqueous core, with diameters typically ranging from 50 to several hundred nanometers. Their amphiphilic architecture confers a unique structural versatility for contrast agent design. Hydrophilic contrast-active species such as Gd^3+^ chelates or iodinated compounds can be encapsulated within the aqueous interior, while hydrophobic agents or lipophilic membrane-intercalating molecules can be incorporated into the bilayer, and targeting ligands or PEG chains can be conjugated to the outer surface [64]. This compartmentalization enables a single liposomal construct to carry a high payload of contrast material per particle, substantially increasing the local concentration of imaging agent at the target site compared to individual small-molecule contrast agents, and thereby improving sensitivity without proportionally increasing the administered dose [64–65].

For MRI applications, Gd^3+^-loaded liposomes have demonstrated relaxivity enhancement arising from both the encapsulated chelate and water exchange dynamics across the lipid bilayer, with the latter representing a tunable parameter, membrane permeability can be adjusted through lipid composition and cholesterol content to optimize the rate of water proton exchange between the interior and the bulk, directly influencing *r*_1_ [65]. For CT, iodinated liposomes have been explored as blood pool agents, exploiting their size to extend vascular retention well beyond the minute-scale window of free iodinated monomers, enabling prolonged angiographic imaging and passive tumor accumulation via the EPR effect [64].

Magnetoliposomes, liposomes encapsulating SPIONs within their aqueous core or intercalated into their bilayer, represent the most extensively investigated liposomal NCA subclass for MRI [66]. By confining multiple iron oxide nanoparticles within a single vesicle, magnetoliposomes achieve collective magnetic behavior and high *r*_2_ relaxivity per particle, making them effective *T*_2_-weighted contrast agents. Beyond imaging, the SPION payload confers magnetic hyperthermia capability when exposed to alternating magnetic fields, enabling theranostic applications in which MRI-guided localization is combined with heat-mediated tumor therapy [66–67]. Magnetoliposomes have additionally been explored as tracers for magnetic particle imaging (MPI); however, as discussed in Section 5.3, MPI performance is highly sensitive to SPION size distribution and interparticle coupling, and the lipid shell introduces additional constraints on core packing density and magnetic relaxation dynamics, which must be carefully managed to preserve signal quality [67].

The biocompatibility profile of liposomes is generally favorable: Phospholipid bilayers are structurally analogous to cell membranes, and PEGylated liposomes exhibit prolonged circulation, reduced opsonization, and low immunogenicity. However, accelerated blood clearance upon repeated dosing, a phenomenon in which anti-PEG IgM antibodies generated after a first injection accelerate hepatic clearance of subsequent doses, has been documented for PEGylated liposomes and represents a translational concern for imaging protocols requiring repeated administration [68]. Encapsulated Gd^3+^ or SPION release upon bilayer disruption in vivo also requires careful evaluation, as premature payload leakage can reduce imaging performance and introduce toxicity risks analogous to those of free small-molecule agents [65]. Despite these challenges, liposomal formulations benefit from a well-established regulatory and manufacturing precedent, several liposomal drug products are clinically approved, which may facilitate the translational pathway for liposomal NCAs relative to entirely novel nanoparticle classes [64].

#### 3.3 Functional and multimodal designs

**3.3.1 Dual-mode NCAs (MRI/CT and *T*_1_/*T*_2_ systems).** Recent work in nanoparticle engineering has increasingly focused on platforms that provide more than one type of imaging signal, such as agents that generate both *T*_1_ (positive) and *T*_2_ (negative) MRI contrast, or those that can support MRI and CT imaging simultaneously. The design of these dual-mode systems is largely guided by three adjustable features. First, the composition of the core, such as mixed Fe/Mn formulations or Gd/Yb-containing structures, allows a single particle to combine magnetic relaxivity with X-ray attenuation. Second, the size and morphology of the nanoparticle shape its *r*_1_ and *r*_2_ relaxivities, and ultimately the *r*_2_/*r*_1_ ratio, which determines whether it functions predominantly as a *T*_1_ or *T*_2_ agent. Third, surface modifications, including PEG layers, silica or protein shells, and targeting ligands, help control circulation time, reduce aggregation, and improve tumor accumulation while supporting predictable in vivo clearance. In MRI/CT hybrid designs, adding high-atomic-number elements such as bismuth or tantalum can significantly enhance X-ray attenuation without necessarily diminishing magnetic performance, although the structural balance of these components must be carefully managed to avoid long-term retention in tissues. Overall, optimizing composition, particle size, and surface chemistry remains central to creating dual-mode NCAs that are realistic candidates for future clinical application [3,46].

**Figure 1:**
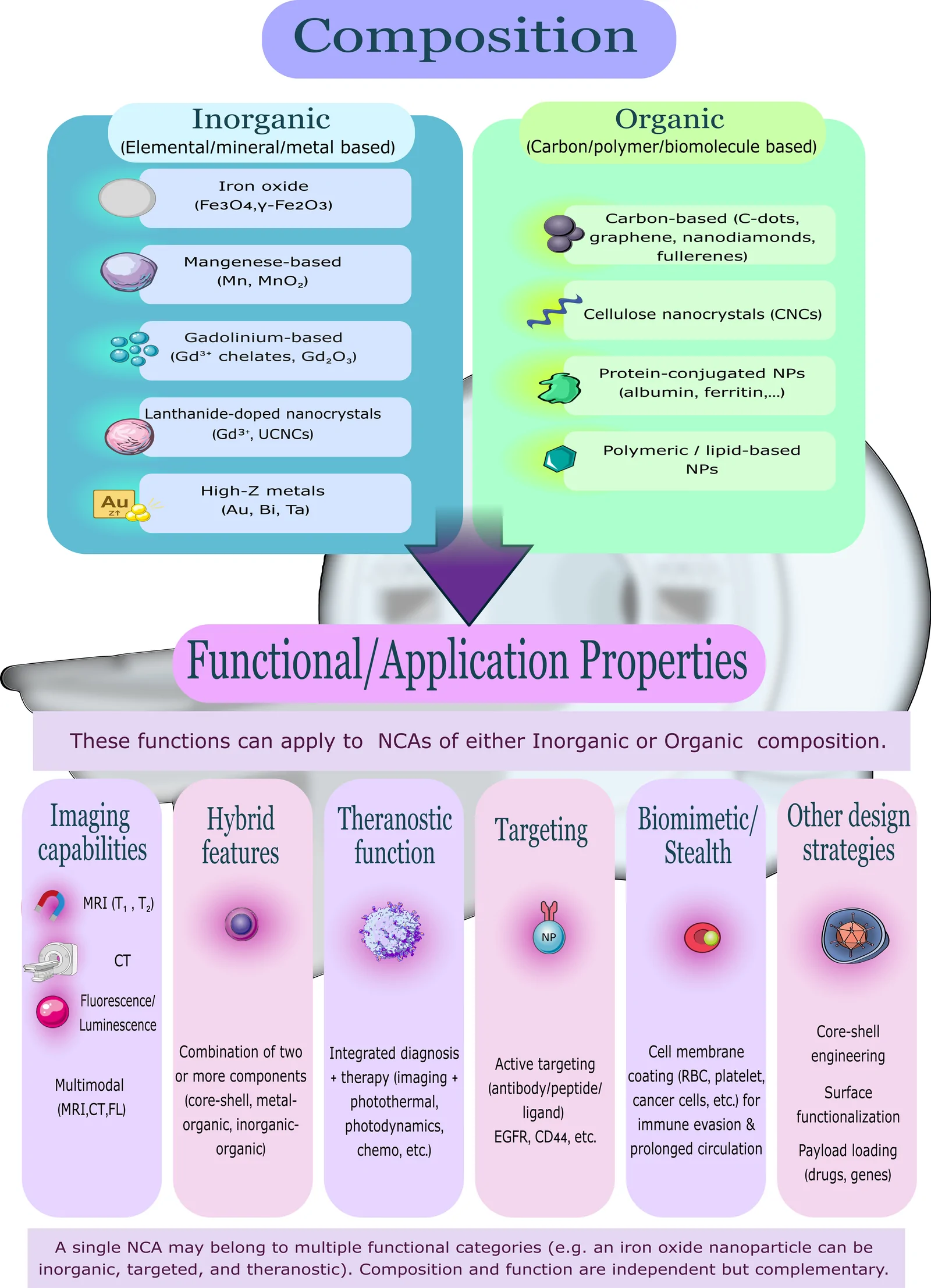
Conceptual classification framework for NCAs used in MRI and CT imaging. (a) Material composition and (b) functional/application properties. A single NCA may belong to multiple functional categories simultaneously. Composition and function are independent but complementary. Scanner Icon: NIAID Visual & Medical Arts. (6/2/2026). Mobile Clinical MRI Scanner. NIAID NIH BIOART Source. https://bioart.niaid.nih.gov/bioart/692. This content is not subject to CC BY 4.0. Theranostic Function Icon: NIAID Visual & Medical Arts. (3/24/2025). B Cell with IgM Receptors. NIAID NIH BIOART Source. https://bioart.niaid.nih.gov/bioart/612. This content is not subject to CC BY 4.0. Lanthanide-based NCA Icon: NIAID Visual & Medical Arts. (9/25/2025). Organoid. NIAID NIH BIOART Source. https://bioart.niaid.nih.gov/bioart/662. This content is not subject to CC BY 4.0. Mangenese-based NCA Icon: NIAID Visual & Medical Arts. (10/9/2025). Organoid. NIAID NIH BIOART Source. https://bioart.niaid.nih.gov/bioart/665. This content is not subject to CC BY 4.0. Core–shell engineering Icon: NIAID Visual & Medical Arts. (5/8/2025). Adenovirus in Vesicle. NIAID NIH BIOART Source. https://bioart.niaid.nih.gov/bioart/647. This content is not subject to CC BY 4.0. RBC Icon: NIAID Visual & Medical Arts. (10/7/2024). Red Blood Cell. NIAID NIH BIOART Source. https://bioart.niaid.nih.gov/bioart/442. This content is not subject to CC BY 4.0. Receptor Icon: NIAID Visual & Medical Arts. (10/7/2024). Receptor Protein. NIAID NIH BIOART Source. https://bioart.niaid.nih.gov/bioart/439. This content is not subject to CC BY 4.0. The remaining icons were adapted from Servier Medical Art (https://smart.servier.com/), licensed under CC BY 4.0 (https://creativecommons.org/licenses/by/4.0/).

**3.3.2 Hybrid and theranostic NCAs.** Hybrid NCAs bring together multiple material components or functional domains within a single construct, allowing for multimodal imaging and, when needed, concurrent therapeutic delivery [69–70]. These systems encompass core–shell architectures, biomimetic nanoparticles, and constructs modified with targeting antibodies [71–72]. A key subset within this category is theranostic nanoparticles, which are explicitly engineered to unite diagnostic and therapeutic capabilities, for example, nanoparticles that combine MRI or CT contrast enhancement with photothermal or photodynamic treatment functions, enabling therapy to be monitored directly through imaging [70–71,73]. Core–shell designs provide flexible control over surface charge, magnetic relaxivity, and drug-loading capacity [74]. Biomimetic coatings derived from red blood cell (RBC) membranes or tumor cell membranes can extend systemic circulation and reduce immune detection [75]. Recent work also demonstrates the compatibility of these hybrid platforms with photon-counting and spectral CT technologies. Incorporating high-atomic-number elements, including gold, bismuth, and tantalum, into the nanoparticle structure enables the system to take advantage of energy-discriminative detection, thereby improving tissue contrast and enhancing diagnostic performance. The clinical relevance of this approach has now been demonstrated in vivo: Spectral photon-counting CT enabled the first reported human gadolinium K-edge images, obtained in a patient who had received a standard clinical dose of a gadolinium-based contrast agent during a prior MRI examination. Notably, gadolinium concentrations that were barely distinguishable from surrounding tissue on conventional CT images were clearly visible on K-edge images, suggesting that clinical doses of gadolinium-based agents may be sufficient for K-edge detection without requiring dedicated, purpose-built contrast formulations. This finding strengthens the translational rationale for Gd-based NCA designs optimized for energy-discriminative CT detection [11]. Taken together, the hybrid and theranostic constructs represent one of the most promising translational directions, pairing advanced imaging precision with therapeutic capability and aligning well with the evolving regulatory landscape for clinically applicable nanotheranostics [76–77]. To provide a unified overview of the structural and compositional diversity of NCAs explored for MRI and CT imaging, their classification system is illustrated in Figure 1.

#### 3.4 Targeting strategies and molecular specificity

The diagnostic specificity of NCAs can be substantially enhanced through active targeting, whereby nanoparticle surfaces are functionalized with ligands that recognize molecular markers selectively expressed or overexpressed at disease sites. This contrasts with the passive accumulation that governs conventional agents and most untargeted NCAs, which rely on the enhanced permeability and retention effect in tumors or nonspecific vascular distribution. Active targeting strategies employ several classes of ligands, each with distinct advantages in terms of size, binding affinity, and conjugation chemistry [4].

Antibodies and antibody fragments provide high binding affinity and specificity for cell-surface antigens. Cetuximab-conjugated gold nanoparticles targeting the epidermal growth factor receptor (EGFR), overexpressed in lung, colorectal, and head-and-neck carcinomas, have demonstrated significantly higher tumor accumulation and CT contrast compared to untargeted particles in xenograft models, with binding affinity shown to be a more decisive determinant of tumor uptake than circulation half-life [78]. Similarly, anti-EGFR peptide-conjugated Gd chelates (EBP-Gd-DO_3_A) have shown superior MRI contrast retention in EGFR-overexpressing tumors compared to non-targeted Gd-DO_3_A, demonstrating that receptor-specific targeting is achievable even with small-molecule Gd frameworks [79].

Peptides offer a smaller, more synthetically accessible alternative to full antibodies, with lower immunogenicity and better tissue penetration. RGD-containing peptides targeting integrin αvβ3, overexpressed on tumor neovasculature, have been conjugated to iron oxide nanoparticles for MRI visualization of angiogenesis. Vascular cell adhesion molecule-1 (VCAM-1)-targeting peptides conjugated to Fe_3_O_4_@SiO_2_ nanoparticles have enabled in vivo MRI detection of atherosclerotic plaque endothelial activation, illustrating the applicability of peptide-targeted NCAs beyond oncology [80].

Aptamers, short single-stranded oligonucleotides selected for high-affinity binding to specific molecular targets, represent a more recent functionalization strategy with particular advantages of low immunogenicity, small size enabling deeper tissue penetration, and facile chemical modification. DNA aptamer M17, selected against matrix metalloproteinase-14 (MMP14) (overexpressed in pancreatic, hepatic, lung, and cervical carcinomas), has been conjugated to SPIONs for targeted *T*_2_-weighted MRI, achieving significantly higher contrast in MMP14-positive cells compared to non-targeted particles [81].

Small molecules including folic acid, hyaluronic acid, and transferrin exploit receptor-mediated uptake pathways that are constitutively overexpressed on rapidly proliferating cells. Folic acid functionalization, targeting the folate receptor overexpressed in ovarian, breast, and colorectal cancers, is already represented in several NCAs described in this review, including NaGdF_4_:Yb^3+^ nanoparticles and gadolinium oxide coated with β-cyclodextrin-polyester functionalized with folic acid (Gd_2_O_3_@PCD-FA) [19,82].

Hyaluronic acid targets CD44, a glycoprotein overexpressed in cancer stem cells and implicated in metastatic potential, as employed in the Prussian blue/FePt nanoparticles coated with hyaluronic acid-grafted PEG (PB@FePt-HA-*g*-PEG) system described in Section 6.2 [83]. The choice of conjugation chemistry is as important as the choice of ligand. Covalent conjugation strategies include carbodiimide coupling between surface carboxyl groups and amine-bearing ligands, thiol–gold bonds for direct attachment of thiol-modified peptides or antibodies to gold nanoparticle surfaces, and click chemistry approaches offering high selectivity and mild reaction conditions. Non-covalent strategies such as streptavidin–biotin linkage offer modular assembly but risk competitive displacement in biological environments [84]. Regardless of strategy, functionalization must be carefully validated to confirm that targeting ligand conjugation does not compromise colloidal stability, alter biodistribution unfavorably, or reduce relaxivity, trade-offs that remain incompletely characterized for most targeted NCA systems reported to date.

### 4 Biocompatibility and safety profiles

#### 4.1 Overview of biocompatibility assessments

The clinical translation of NCAs depends heavily on their biocompatibility and safety. These factors are shaped by nanoparticle composition, surface modification strategies, and clearance mechanisms [6]. In addition to standard in vitro assessments, clinical translation requires reproducible and scalable synthesis methods, well-characterized and quantifiable clearance pathways (renal versus hepatobiliary), and standardized safety evaluations, including hemolysis, complement activation, coagulation profiles, and cytokine responses. Comparative benchmarking against approved clinical contrast agents, such as Gd-DTPA or iohexol, and early human pharmacokinetic studies represent essential de-risking steps that are increasingly recognized as prerequisites for regulatory approval [3,46].

Here, benchmarking refers to the direct head-to-head evaluation of an investigational NCA against an approved clinical contrast agent, such as Gd-DTPA for MRI or iohexol for CT, under matched experimental conditions, measuring parameters including relaxivity or X-ray attenuation, pharmacokinetic profile, and acute safety endpoints; the approved agents serve as the established reference standard against which the investigational agent’s performance and safety must be shown to be superior or non-inferior before regulatory bodies will consider its advancement to clinical trials [3,20–21,58]. The following section provides an overview of biosafety data across major categories of MRI/CT NCAs.

**4.1.1 Regulatory considerations for clinical translation and approval.** The transition of novel NCAs from preclinical validation to clinical imaging requires navigating stringent regulatory frameworks managed by the U.S. Food and Drug Administration (FDA) and the European Medicines Agency (EMA). To secure clinical approval, developers must establish rigorous preclinical toxicology profiles under “good laboratory practice” (GLP) guidelines, utilizing independent biological replication to verify batch-to-batch consistency. Required assessments include comprehensive physicochemical characterization, in vitro cytotoxicity, hemocompatibility, and immunogenicity profiling. Crucially, in vivo pharmacokinetic and biodistribution assays must delineate clear clearance pathways, assessing whether the nanomaterials undergo safe renal excretion or suffer from long-term hepatic accumulation. Following preclinical validation, these agents must undergo standard, multiphase human clinical trials to conclusively demonstrate superior or non-inferior diagnostic efficacy and safety compared to existing commercial contrast agents. These trials progress sequentially from phase-I studies, which assess early human safety, tolerability, and preliminary pharmacokinetics in small cohorts, through phase-II and phase-III studies that evaluate diagnostic efficacy, optimal dosing, and comparative performance against approved agents in progressively larger patient populations [85–86]. This translational pipeline represents a major developmental bottleneck, requiring significant financial investment and over a decade of development before a novel NCA can be integrated into routine clinical imaging [87–88].

A critical component of this pipeline is the transition to “good manufacturing practice” (GMP)-compliant production, which requires that synthesis processes be fully documented, validated, and reproducible at scale under controlled conditions that satisfy regulatory expectations for clinical-grade nanomaterials. Translational barriers for NCAs extend beyond manufacturing and encompass four interconnected challenges. First, manufacturing challenges remain significant: Even when small-scale synthesis yields nanoparticles with excellent imaging performance and biocompatibility, replicating these properties consistently at GMP-compliant production scale, with batch-to-batch reproducibility and rigorous quality control, has proven difficult for most NCA classes and represents one of the primary reasons investigational agents fail to advance beyond preclinical stages [89–90]. Second, regulatory challenges are substantial: Unlike small-molecule drugs, NCAs do not fit neatly into existing regulatory frameworks, and the absence of harmonized guidance specifically addressing inorganic nanomedicines as a distinct product class means that developers must negotiate the required preclinical data package with regulatory agencies on a case-by-case basis, adding time and uncertainty to the approval pathway [85,91]. Third, cost and time barriers are formidable: The full translational pipeline from initial nanoparticle design to clinical approval typically requires over a decade of development and hundreds of millions of dollars in investment, a threshold that has limited serious clinical translation efforts to a small number of well-resourced academia–industry partnerships [85–86]. Fourth, standardization challenges undermine cross-study comparisons and slow collective progress: The absence of uniform preclinical testing protocols, inconsistent physicochemical characterization reporting, and variability in relaxivity measurement conditions across laboratories make it impossible to reliably rank NCA candidates against one another or against approved clinical agents, and community-wide harmonization efforts are urgently needed [3–4,92].

Physicochemical optimization is central to navigating this pipeline successfully. Size is the most consequential parameter. Nanoparticles must be engineered within a narrow window, large enough to avoid premature renal filtration that truncates circulation time, yet small enough to evade sustained hepatosplenic sequestration that causes long-term organ retention and toxicity. For most NCA classes, this translates to a target hydrodynamic diameter of 6–50 nm, with the lower bound set by the glomerular filtration threshold and the upper bound by mononuclear phagocyte system recognition efficiency [26–27]. Morphology also matters as rod-shaped and plate-like particles exhibit differential circulation times and cell uptake kinetics compared to spheres of equivalent volume, a parameter that remains underexplored in the NCA literature [31]. Surface chemistry is arguably the most versatile handle. PEGylation reduces opsonization and extends circulation, zwitterionic coatings enable renal clearance while maintaining colloidal stability, and biomimetic coatings such as red blood cell membranes reduce immune recognition [24–25]. The core composition determines relaxivity or X-ray attenuation but must be balanced against ion leakage risks, particularly for Gd^3+^ and Mn^2+^, which depend on ligand or coating integrity under the acidic and reducing conditions of endosomes and the tumor microenvironment. Collectively, these parameters are interdependent; optimizing one frequently perturbs another, and the path to clinical translation requires their simultaneous co-optimization rather than sequential tuning, a challenge that has contributed to the limited number of NCAs that have reached regulatory approval to date [85].

**4.1.2 Clearance mechanisms of NCAs.** Renal clearance, the most desirable elimination route, operates through glomerular size-selective filtration as described in Section 2, with a hydrodynamic diameter threshold of 5.5–6.0 nm governing efficient urinary excretion [26–27]. Surface charge and protein corona formation can shift this threshold significantly, making neutral or zwitterionic coatings a prerequisite for reliable renal clearance [26]. Nanoparticles with hydrodynamic diameters between 6 and 200 nm that escape renal filtration are predominantly processed by the hepatobiliary route. Upon reaching the liver via the systemic circulation, these particles are taken up by Kupffer cells, the resident macrophages lining the hepatic sinusoids, as well as by liver sinusoidal endothelial cells, and are subsequently transferred into the bile for excretion via the intestinal tract and feces. This process occurs over a timescale of hours to weeks, depending on particle properties and degradability [93]. Non-degradable particles that resist biliary processing may be retained in hepatic tissue for extended periods, raising the risk of chronic toxicity, particularly at the doses required for repeated imaging [93–94].

The mononuclear phagocyte system (MPS), comprising macrophages and monocytes distributed throughout liver, spleen, bone marrow, and lymph nodes, represents the third and most problematic clearance route from a translational perspective. Opsonization, the adsorption of complement proteins and immunoglobulins onto the nanoparticle surface, triggers recognition and phagocytic uptake by MPS cells, leading to accumulation predominantly in liver and spleen [94]. Particles exceeding approximately 200 nm are particularly susceptible to splenic mechanical filtration through the interendothelial slits of the red pulp, in addition to MPS-mediated phagocytosis. Non-degradable particles sequestered by the MPS may persist in these organs for months to years, making MPS accumulation a critical long-term safety concern for NCAs that cannot be efficiently cleared by renal or biliary routes [93–95].

The interplay between these three pathways defines a practical size window for NCA design. Particles below approximately 6 nm achieve rapid renal clearance but may suffer from insufficient circulation time for target accumulation. Particles in the 6–200 nm range can achieve prolonged vascular residence suitable for tumor imaging via the EPR effect or active targeting, but require careful surface engineering to limit MPS recognition and eventual hepatosplenic retention. Particles above ca. 200 nm face rapid splenic filtration and are generally unsuitable for systemic NCA applications. Surface modification strategies, including PEGylation, zwitterionic coatings, and biomimetic membrane camouflage, are employed across all size ranges to modulate protein corona formation, extend circulation, and shift the clearance balance toward safer renal or biliary routes, as discussed in Section 4.3 [26–27,93].

#### 4.2 Biosafety by material class

To properly contextualize the safety and performance of investigational NCAs, it is essential to consider the clinically approved agents that currently serve as gold standards. For MRI, GBCAs have dominated clinical practice since the late 1980s. Gadopentetate dimeglumine (Magnevist^®^), a linear ionic Gd^3+^-DTPA chelate, was the first approved agent and remains widely used for central nervous system (CNS) and vascular imaging; it is excreted renally and produces *T*_1_-positive contrast through its seven unpaired electrons and long electronic relaxation time, with a typical *r*_1_ of approximately 3–5 mM^−1^·s^−1^ at 1.5 T [58,96]. Linear GBCAs such as Magnevist^®^ are, however, associated with NSF in patients with renal impairment and with gadolinium deposition in the brain and bone following repeated administration, risks that are substantially lower with macrocyclic agents such as gadobutrol (Gadovist^®^), whose 1,4,7,10-tetraazacyclododecane-1,4,7,10-tetraacetic acid (DOTA)-based macrocyclic ligand confers higher thermodynamic and kinetic stability and a markedly reduced rate of free Gd^3+^ release [58,97].

For CT, iohexol (Omnipaque^®^), a low-osmolar nonionic iodinated monomer, represents the most widely administered conventional agent, providing X-ray attenuation through its high iodine content; its principal limitations are contrast-induced nephropathy in at-risk patients and hypersensitivity reactions ranging from mild urticaria to anaphylaxis [20–21].

**4.2.1 Gd-based nanoparticles.** Common Gd-based NCAs include Gd-doped mesoporous carbon nanoparticles (Gd-MCNs), fluorescent silica-coated Gd nanodots (FS-GdNDs), polytungstate nanoclusters, and Gd-chelating pullulan nanogels (Gd-CHPOA) [32,98–99]. These agents have demonstrated highly biocompatibility, low reactive oxygen species (ROS) generation, limited cytokine production, and negligible cytotoxicity in vitro and in vivo [32,72,100–101]. Compared to conventional Gd-DTPA, Gd-MCNs show minimal RBC distortion and protein denaturation, supporting their cellular compatibility and safety [32]. This group of contrast agents showed low toxicity, efficient renal clearance, high blood compatibility, and no apparent tissue damage in main organs, indicating good biocompatibility and safety for practical applications [102]. In one study, Zhang et al. introduced Gd-MCNs as a clinical MRI-NCA. The results demonstrated excellent hemocompatibility (hemolysis < 3%) and cellular safety. In vivo studies revealed no significant adverse effect to organs and hematological factors. Compared to Gd-DTPA, Gd-MCNs caused lower ROS production, reduced oxidative stress, and minimal secretion of inflammatory factors. Notably, no DNA damage was observed. Gd-MCNs were predominantly excreted via the hepatobiliary system rather than the kidneys, suggesting a reduced risk of nephrotoxicity [32].

Another agent, Gd_2_O_3_@PCD-FA, was developed by coating Gd_2_O_3_ NCAs with β-cyclodextrin (CD)-based polyester (PCD) and functionalizing them with folic acid (FA). At concentrations up to 50 μg·mL^−1^, no significant cytotoxicity was observed, whereas uncoated Gd_2_O_3_ displayed notable cytotoxicity, likely due to Gd^3+^ ion leakage (*p* < 0.005). Across concentrations ranging from 1.95 to 500 μg Gd^3+^·mL^−1^, hemolytic activity remained below 6%, within the generally accepted safety threshold. Hemolysis was significantly lower for Gd_2_O_3_@PCD-FA compared to uncoated Gd_2_O_3_, indicating that the PCD surface coating markedly enhances biosafety and mitigates the toxicity associated with unmodified Gd_2_O_3_ [19].

Finally, Gd-CHPOA was synthesized from cholesterol- and acryloyl-modified polysaccharide pullulan and Gd^3+^. At 1 day, 1 week, 1 month, and 3 months after injection of the nanogel, no histopathological abnormalities were noted in liver or spleen tissue. No evidence of swelling, necrosis, or inflammation of hepatocytes was noted. Serum biochemistry remained within normal limits, and no signs of systemic toxicity were observed, indicating excellent long-term biocompatibility [72].

In another approach, 25 nm Gd^3+^-doped amorphous TiO_2_ nanoparticles (GTNs) showed minimal effects on blood coagulation, as both prothrombin time and activated partial thromboplastin time remained within the normal range, suggesting that these nanoparticles do not significantly interfere with the extrinsic or intrinsic coagulation cascades. Nanoparticle-treated samples exhibited no signs of hemolysis, even at a high concentration of 200 μg·mL^−1^, thereby supporting the non-hemolytic profile of GTN NCAs. Neither the undoped titania (TN) nor the GTN variants induced significant changes in platelet counts, suggesting that platelet aggregation was not stimulated. Furthermore, both TN and GTN nanoparticles failed to elicit notable elevations in pro-inflammatory cytokines, including IL-1β, IL-8, IL-10, and IL-12p70, which indicates a minimal inflammatory response. Additionally, neither TN nor GTN demonstrated lactate dehydrogenase (LDH) release or cytotoxic effects after a 24 hour incubation period. Intravenous administration of these agents is a safe option for imaging purposes in humans without any adverse reactions [103].

**4.2.2 Iron-containing nanoparticles.** Iron-based nanoparticles include zwitterion-coated ultrasmall iron oxide nanoparticles (ZUIONs), SPIONs (Fe_2_O_3_/Fe_3_O_4_), FePt nanoparticles, and dextran-coated SPIONs (SPIONDex) [28,104–108]. The in vivo behavior of iron-containing nanoparticles is strongly influenced by their hydrodynamic size, surface charge, and coating composition, which collectively determine protein corona formation, mononuclear phagocyte system recognition, circulation half-life, and the balance between renal and hepatosplenic clearance [93].

Two SPION formulations reached regulatory approval for hepatic MRI, namely, ferumoxides (Feridex^®^/Endorem^®^), composed of dextran-coated iron oxide nanoparticles of 120–180 nm, and ferucarbotran (Resovist^®^), consisting of carboxydextran-coated iron oxide cores of approximately 60 nm, both exploiting *T*_2_/*T*_2_* signal loss in healthy hepatic parenchyma to delineate focal lesions [36]. Despite their diagnostic utility, both were withdrawn from most markets due to acute hypersensitivity reactions, manufacturing instability, and suboptimal relaxivity [108].

Ferumoxytol (Feraheme^®^), a polyglucose sorbitol carboxymethylether-coated SPION of approximately 30 nm, currently represents the only iron-based agent available for intravenous clinical use, approved for iron deficiency anemia and used off-label as a blood-pool MRI agent owing to its prolonged vascular residence and absence of gadolinium-related nephrotoxicity risk; however, it carries an FDA boxed warning for serious hypersensitivity reactions, underscoring that even the most advanced approved iron-based agents have not fully resolved the safety limitations that NCAs aim to overcome [109]. These benchmarks collectively define the safety and performance bar that NCAs must meet or exceed to justify clinical translation, as further discussed in the material-specific sections below and summarized below in Table 3.

ZUIONs exhibited minimal hemolysis, low cytotoxicity, and no detectable tissue injury in major organs following injection in mice. They remained structurally stable in vivo and were predominantly cleared via the renal pathway, suggesting excellent clinical safety potential [104]. Fe_2_O_3_/Fe_3_O_4_ nanoparticles showed favorable biosafety profiles, including absence of kidney injury, normal hematological and serum chemistry indices, and preserved liver function markers. They underwent efficient metabolic clearance and exhibited water solubility, biocompatibility, and colloidal stability [105–107,110–113]. Adequate biodegradation may help mitigate long-term nanomaterial-related biological uncertainties [114–115]. Bovine serum albumin (BSA)-functionalized Fe_2_O_3_ nanoparticles (Fe_2_O_3_@BSA) were synthesized via a protein-templated in vitro approach, distinct from biological biomineralization by magnetotactic bacteria, in which BSA self-assembled into a cage-like nanocage structure that directed iron oxide nucleation and crystal growth, simultaneously conferring colloidal stability and a biocompatible protein shell [39].

These nanoparticles demonstrated strong *T*_1_/*T*_2_ MRI performance alongside a favorable short-term biosafety profile in preclinical models. In vitro, no cytotoxicity was observed in live/dead assays at iron concentrations up to 300 μg·mL^−1^. In vivo, blood biochemical parameters and hematoxylin and eosin (H&E)-stained histology of major organs showed no abnormalities at 7 and 14 days post-injection compared to controls, though it should be noted that this observation window is relatively short and does not address potential chronic effects of long-term organ retention. Assessments of hemocompatibility, complement activation, and immune responses were not reported in the primary source, representing gaps in the available biosafety profile. The favorable biocompatibility relative to uncoated iron oxide nanoparticles was attributed to the shielding and stabilizing effects of the BSA shell, which reduces non-specific protein interactions and limits ion leaching [39,116–118].

SPIONDex, a SPION formulation coated with cross-linked dextran, demonstrated favorable cyto-, hemo-, and immunocompatibility in direct head-to-head in vitro comparisons with the clinically approved agents ferucarbotran and ferumoxytol, as detailed below. Compared with ferucarbotran and ferumoxytol, SPIONDex did not activate complement, induce pseudoallergic reactions, or impair either the intrinsic or extrinsic coagulation pathways. In contrast, ferumoxytol and ferucarbotran strongly activated the complement system in porcine models [119], induced robust complement activation and pseudoallergic reactions [120], and triggered complement activation-related pseudoallergy (CARPA) even at low doses [108].

Regarding cytotoxicity, ferucarbotran exhibited significant cytotoxicity in Jurkat cells (a T-cell leukemia line), whereas SPIONDex demonstrated no cytotoxicity in human macrophages, endothelial cells, or THP-1 monocytes (a human monocytic leukemia cell line). However, because these assessments were conducted in different cell types rather than under matching experimental conditions, a direct quantitative comparison of cytotoxic potency between the two agents is not straightforward and should be interpreted with caution [36,121–123]. The absence of SPIONDex from the clinic despite its favorable preclinical profile reflects broader barriers to SPION translation, including the requirement for large-scale manufacturing with demonstrated batch-to-batch reproducibility, comprehensive long-term toxicology packages extending beyond the short observation windows used in preclinical studies, and the absence of harmonized regulatory guidance specifically addressing iron oxide nanomedicines as a distinct product class [108,124]. Additionally, SPIONDex did not affect platelet activation, further supporting its favorable immunocompatibility profile [108].

**4.2.3 Mn-Based nanoparticles.** Mn-based nanoparticles, including manganese silicate nanospheres (MnSNs) and manganese dioxide poly(acrylic acid) nanoparticles (MnO_2_/PAA), have demonstrated low systemic toxicity, efficient renal clearance, and no histological abnormalities. Mn is rapidly cleared from heart, liver, spleen, and lungs within 24 h, and within 72 h from the kidneys, showing minimal tissue retention [54,125–127]. The potential toxicity of Mn-based nanoparticles is modulated by physicochemical parameters including particle size, surface coating, and surface charge, which collectively influence cellular uptake, iron/metal ion release, and oxidative stress induction. Surface functionalization plays a particularly important role; appropriate coatings reduce non-specific interactions, limit ion leakage under acidic endosomal conditions, and can significantly attenuate cytotoxic responses compared to bare nanoparticles. Evaluation of Mn-based nanoparticle safety typically employs standard in vitro assays, such as tetrazolium-based cell viability tests, LDH release, and ROS quantification, alongside in vivo hematological and histopathological analyses, which together provide a comprehensive toxicological profile [94].

**4.2.4 Carbon-based nanoparticles.** Among carbon-based NCAs, a representative theranostic example is the aggregated C-dots system (A-C-dots@Ce6), where “A” denotes the assembled or aggregated state of the discrete C-dot building blocks. In this system, negatively charged C-dots are cross-linked into larger assemblies using Gd^3+^ ions as electrostatic “glue”; the incorporated Gd^3+^ ions are the direct source of *T*_1_-weighted MRI contrast, shortening longitudinal relaxation times in a manner analogous to conventional Gd-based chelates. The photosensitizer Ce6 was loaded onto these assemblies to enable photodynamic therapy and fluorescence imaging, making A-C-dots@Ce6 a multimodal theranostic platform rather than a purely diagnostic contrast agent. The assembly strategy simultaneously addresses a key pharmacokinetic limitation of ultrasmall C-dots, their short vascular circulation time due to rapid renal clearance, by increasing the hydrodynamic diameter of the construct to allow for sufficient tumor accumulation before eventual clearance. A-C-dots@Ce6 demonstrated negligible cytotoxicity even at high concentrations alongside dual fluorescence and MRI imaging capability in preclinical models [128]. Optimizing carbon-derived NCAs requires careful manipulation of surface chemistry and electronic structure. Doping with heteroatoms, surface passivation, and control of particle aggregation markedly influence relaxivity, fluorescence stability, and biodistribution. Tailoring carboxyl, amine, or PEG groups has proven essential for achieving both strong imaging contrast and low toxicity [129].

The pharmacokinetic behavior of carbon-based NCAs is strongly size-dependent, mirroring the general renal clearance threshold established for nanoparticles in Section 2. Carbon dots below approximately 6 nm undergo predominantly renal excretion, while those exceeding 8 nm are more likely to follow hepatic processing routes with a higher risk of bioaccumulation. In multiple carbon dot-containing (multi-CD) nanoparticle formulations, hepatobiliary excretion has been confirmed in vivo, with clearance through intestinal feces. Toxicity profiles of CDs are highly context-dependent, influenced by synthesis method, surface charge, and chemical composition rather than carbon core properties alone, underscoring the importance of per-formulation characterization over generalized class-level safety assumptions [57,130].

Despite the encouraging biosafety data summarized above, several critical gaps undermine the strength of conclusions that can currently be drawn across NCA material classes. First, the overwhelming majority of biocompatibility assessments rely on short-term in vitro assays, that is, MTT, LDH release, and hemolysis, performed under idealized conditions that do not replicate the complexity of the in vivo environment, including protein corona dynamics, immune cell interactions, and organ-specific accumulation over time. The disconnect between in vitro and in vivo outcomes is well established for nanoparticulate systems, and the NCA literature is not exempt from this limitation [4]. Second, the absence of standardized testing protocols across studies makes direct cross-material comparisons unreliable; cell lines, exposure durations, dose ranges, and endpoints differ substantially between laboratories, meaning that a favorable biocompatibility profile reported for one NCA formulation cannot be meaningfully ranked against another without methodological harmonization [7]. Third, and most critically, long-term biodistribution and clearance data beyond 30 days are available for very few NCA classes, and chronic organ retention, particularly hepatosplenic accumulation of particles exceeding the renal filtration threshold, remains largely uncharacterized at the doses and timescales relevant to repeated clinical use [94]. Addressing these gaps through standardized preclinical pipelines and longer-term in vivo studies in relevant animal models is a prerequisite for credible regulatory submissions, not an optional refinement.

#### 4.3 Surface engineering strategies to enhance biosafety

**4.3.1 PEGylation.** The attachment of PEG to other compounds, drugs, or contrast agents is a widely used method aimed at improving safety and enhancing efficacy. PEGylated molecules typically exhibit lower toxicity, reduced antigenicity and immunogenicity, and increased water solubility, thereby improving clinical utility. For instance, PEG-modified Gd nanoparticles have been used as vascular contrast agents in micro-CT. These PEG conjugates not only offer better tolerance but also enhance the pharmacokinetic profile, improve molecular penetration, and provide additional protection against proteolytic enzymes [131–133]. While PEGylation offers numerous advantages in the development of contrast agents, potential challenges must be considered, especially in patients with known or suspected allergies to PEG. Future research and development should aim to address these challenges in order to enhance safety and efficacy of PEGylated contrast agents [134].

**4.3.2 Red blood cell-membrane-camouflaged nanoparticles.** RBC membrane-camouflaged nanoparticles are fabricated by coating synthetic nanoparticle cores with plasma membrane vesicles derived from autologous or allogeneic erythrocytes. The coating process typically involves hypotonic lysis of RBCs to obtain membrane ghosts, followed by co-extrusion or sonication of the membrane material with the nanoparticle core to produce a right-side-out lipid bilayer shell that preserves native membrane proteins including CD47, the “don’t-eat-me” signal that inhibits macrophage phagocytosis, and glycophorin A, which confers long-circulating properties analogous to those of endogenous erythrocytes [135]. This biomimetic strategy confers immune evasion without the anti-PEG antibody concerns associated with PEGylation, and allows for flexible surface modification of the inner core independently of the outer membrane shell. RBC membrane-camouflaged nanoparticles demonstrate favorable biocompatibility, supporting their potential as a biomimetic platform for future clinical applications. Although further research is needed to systematically assess short- and long-term toxicity at various dosages, initial small-scale studies provide a foundation for exploring these biomimetic nanomaterials in biomedical applications [136].

**4.3.3 Cellulose nanocrystals.** Cellulose nanocrystals (CNCs) are rod-shaped biodegradable nanoparticles derived from plant or bacterial cellulose via acid hydrolysis, with dimensions typically in the range of 5–20 nm in width and 100–500 nm in length [137]. Their surface hydroxy groups provide a versatile handle for chemical modification, and their inherently low toxicity and renewable origin make them attractive as biocompatible NCA scaffolds [138]. For MRI applications, CNCs have been used as scaffolds for Gd-based contrast materials, with Gd_2_O_3_ nanoparticles loaded onto PEGylated CNC platforms producing dual *T*_1_/*T*_2_ contrast with high relaxivity values and no cytotoxicity in HeLa human cervical cancer cell lines [139]. For CT, the option to load high-atomic-number dopants onto the CNC surface extends their utility to X-ray attenuation contrast. Mn^2+^ anchored onto DTPA-functionalized CNCs has also demonstrated enhanced MRI contrast with *r*_1_ values up to 57.71 mM^−1^·s^−1^ alongside favorable biocompatibility [54]. The biosafety of both unmodified and surface-modified CNCs in vitro supports their potential as dual MRI and CT contrast platforms [140].

**4.3.4 Copper-based nanoparticles.** This group of NCAs includes copper zinc tin sulfide (CZTS) nanoparticles conjugated with BSA, which exhibit good biocompatibility and low cytotoxicity. In the original in vivo study, intravenously administered CZTS-BSA nanoparticles produced no detectable hepatotoxicity, nephrotoxicity, or hematological abnormalities at imaging-relevant doses, with serum biochemistry and histological evaluation of major organs remaining within normal limits throughout the observation period [141]. These findings are consistent with the broader pattern observed across multiple NCA classes: In vivo studies of PEGylated ZUIONs showed no organ injury and normal hematological indices following intravenous injection in rodents [104], Mn-based MnSNs and MnO_2_/PAA nanoparticles showed clearance from major organs within 24–72 h with no histological abnormalities [125–127], and Gd-CHPOA nanogels produced no hepatopathological findings at 1 day, 1 week, 1 month, or 3 months post-injection [72].

By contrast, the gold-standard Gd-based chelates, particularly linear agents such as Magnevist^®^, are associated with NSF in renally impaired patients and with progressive gadolinium deposition in the brain and bone following repeated administration [58,96]. Conventional iodinated agents carry risks of contrast-induced nephropathy and hypersensitivity reactions ranging from urticaria to anaphylaxis [20–21,142]. The approved iron oxide agent ferumoxytol carries an FDA boxed warning for serious hypersensitivity reactions [109]. Across these comparisons, the available preclinical evidence suggests that well-engineered NCAs produce fewer acute adverse effects than their conventional counterparts, though this conclusion must be tempered by the scarcity of long-term and human safety data for most investigational formulations.

**4.3.5 Additional surface modification strategies.** Beyond PEGylation and biomimetic membrane coatings, several other surface modification strategies have been developed to address specific limitations in NCA performance and safety. Zwitterionic coatings, bearing equal numbers of positively and negatively charged functional groups within the same molecule, such as carboxybetaine, sulfobetaine, or phosphocholine derivatives, offer an alternative to PEG that resists non-specific protein adsorption through tightly bound hydration layers, avoids the anti-PEG immune response, and in the ultrasmall size regime can enable efficient renal clearance while maintaining colloidal stability, as demonstrated by ZUIONs [104,143]. Silica shells represent another widely used surface strategy; a thin amorphous SiO_2_ layer deposited around a magnetic or metal core provides chemical inertness, protects the core from ion leaching in biological fluids, reduces non-specific binding, and presents a surface rich in hydroxy groups for further conjugation of targeting ligands or therapeutic molecules, as demonstrated in silica-coated iron/iron oxide (Fe@FeOx@SiO_2_) core–shell NCAs [30]. Dextran and its derivatives, as used in the clinically approved ferucarbotran and in SPIONDex, coat the iron oxide surface with a hydrophilic polysaccharide layer that reduces complement activation and mononuclear phagocyte system recognition while supporting colloidal stability across a range of physiological conditions [108]. Hyaluronic acid functionalization serves a dual role: As a hydrophilic stabilizing coating it improves biocompatibility, but more distinctively it actively targets CD44 receptors overexpressed on many tumor and cancer stem cells, enabling receptor-mediated endocytosis and selective tumor accumulation without requiring additional targeting ligands [144]. Finally, small-molecule targeting ligands, including folate for folate-receptor-positive tumors, arginine–glycine–aspartic acid (RGD) peptides for integrin-expressing tumor vasculature, and anti-EGFR antibody fragments, can be conjugated to any of the above surface platforms to superimpose active targeting specificity onto the passive pharmacokinetic improvements that the base coating provides [19,72,143]. The choice among these strategies is ultimately governed by the specific translational requirements of the type of NCA; clearance route, target tissue, imaging modality, manufacturing scalability, and regulatory precedent each impose constraints that make a universal optimal coating unlikely and individualized co-optimization of core and surface the defining challenge of NCA design.

#### 4.4 Determinants and outlook for biocompatible CA design

The field of biocompatible contrast agents is rapidly advancing and holds significant promise for enhancing the safety and effectiveness of biomedical imaging. Biocompatible NCAs offer improved safety profiles, reduced systemic toxicity, and enhanced imaging performance, positioning them as next-generation alternative to conventional agents [1,145]. Biocompatible NCAs not only enhance MRI and CT performance but also rectify the biocompatibility issues associated with traditional contrast agents. With continued innovation, the clinical adoption of NCAs is expected to grow, revolutionizing the field of medical imaging [23].

In oncology, targeted NCAs have demonstrated meaningful improvements in tumor visualization and treatment planning beyond what conventional agents achieve. Actively targeted formulations, such as anti-EGFR antibody-conjugated gold nanoparticles and folate-receptor-targeted Gd-based nanoparticles, achieve tumor-specific accumulation through receptor-mediated uptake that passive small-molecule agents cannot replicate, enabling earlier detection and sharper delineation of tumor margins [124]. Theranostic NCAs further integrate diagnostic contrast with therapeutic payload delivery, allowing for real-time imaging-guided therapy that is impossible with conventional contrast agents [70,73]. Regarding lymph node staging, a critical determinant of cancer prognosis and treatment selection, iron oxide NCAs have been particularly well studied. Ultrasmall superparamagnetic iron oxide (USPIO) particles, following intravenous administration, are taken up by macrophages in normal lymph nodes, causing focal *T*_2_* signal loss on MRI; metastatic nodes, which lack functional macrophages, fail to accumulate the particles and therefore remain signal-bright, creating a high-contrast distinction between benign and malignant nodes that size-based criteria on conventional MRI and CT cannot achieve [146]. This capability is especially relevant in prostate, bladder, and colorectal cancers, where accurate nodal staging directly determines eligibility for curative surgery versus systemic therapy. Clinical studies using ferumoxtran-10 (Combidex^®^) demonstrated detection of lymph node metastases, including nodes of normal size that were missed entirely by conventional imaging, with significantly higher sensitivity than standard MRI [147–148].

The safety profile of NCAs, while still requiring long-term human validation, compares favorably with conventional agents across several well-documented adverse event categories. Acute hypersensitivity reactions, the most clinically significant concern with iodinated agents and ferumoxytol, have been reported at low or negligible rates with investigational NCAs across multiple material classes: Hemolysis remained below 3–6% for Gd-based NCAs [19,32], complement activation, the mechanism underlying the pseudoallergic reactions seen with ferumoxytol and ferucarbotran in porcine models [119–120], was absent with SPIONDex [108], and no coagulation pathway interference was detected with Gd-doped TiO_2_ nanoparticles [103].

Stimuli-responsive NCAs represent a further advance in this direction; by remaining inert in healthy tissue and releasing their contrast-active components only in pathological microenvironments, such as the mildly acidic, glutathione-rich milieu of solid tumors in the case of MnO_2_-based systems [54–56], they concentrate imaging signal at the site of disease while minimizing systemic exposure to active metal ions, an approach that structurally reduces the risk of off-target toxicity rather than relying solely on passive clearance [149–150]. With continued advances in nanotechnology and materials science, next-generation NCAs are also expected to deliver higher spatial resolution and improved environmental responsiveness, including signal modulation triggered by enzymatic activity or ROS [149,151]. Furthermore, integrating artificial intelligence into NCA-based imaging can enable faster, more accurate diagnoses by detecting subtle patterns often missed by human interpretation [151].

**Figure 2:**
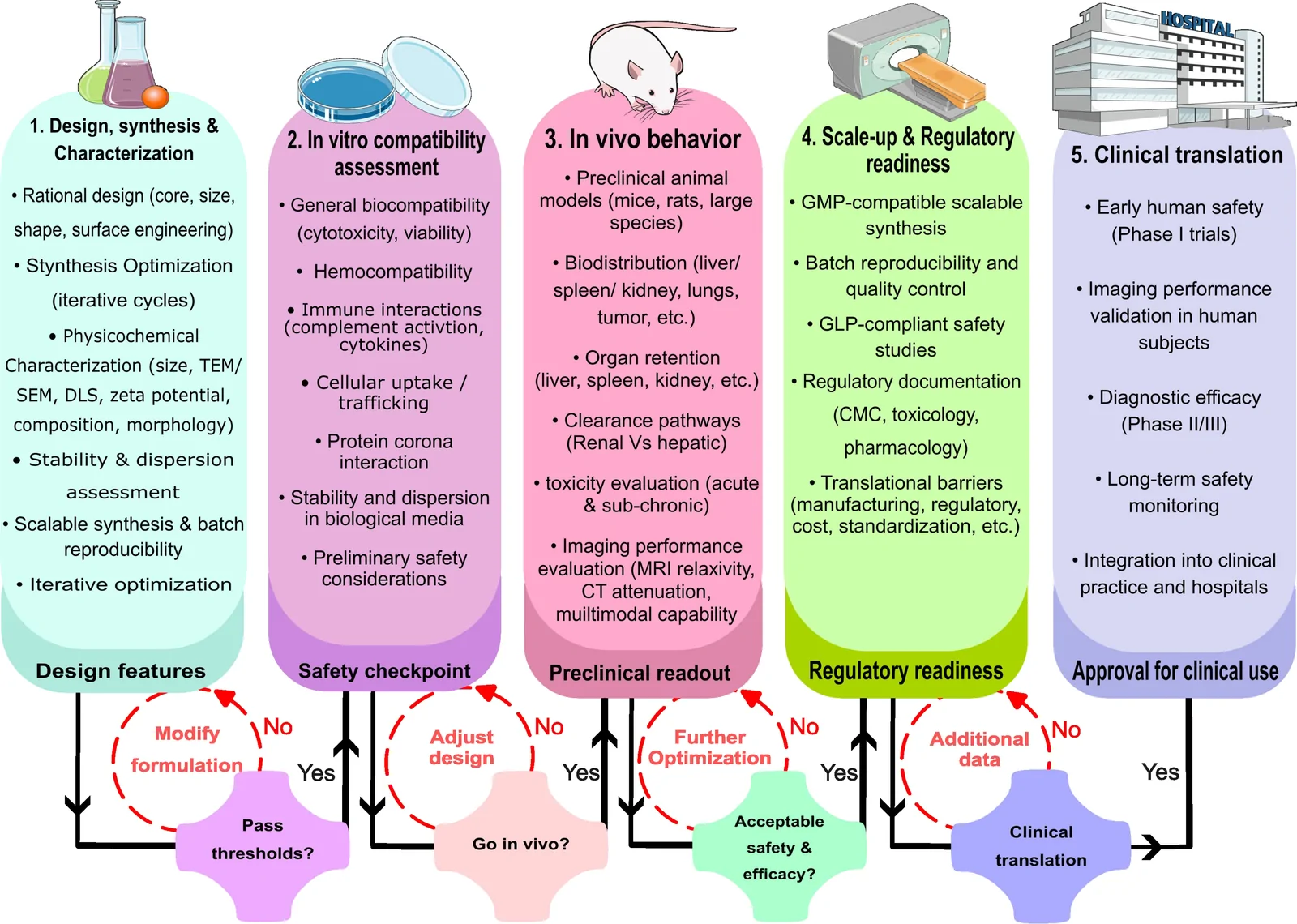
Translational workflow of nano contrast agents (NCAs) from design and synthesis to clinical application. (1) Design, synthesis and characterization, (2) in vitro compatibility assessment, (3) in vivo behavior, (4) scale-up and regulatory readiness, and (5) clinical translation. Lab Mouse Icon: NIAID Visual & Medical Arts. (10/7/2024). Lab Mouse. NIAID NIH BIOART Source. https://bioart.niaid.nih.gov/bioart/283. This content is not subject to CC BY 4.0. The remaining icons were adapted from Servier Medical Art (https://smart.servier.com/), licensed under CC BY 4.0 (https://creativecommons.org/licenses/by/4.0/).

Even though biocompatible MRI and CT scan NCAs are generally safer than conventional alternatives, they might nevertheless have negative biological effects. These effects, including NSF, organ-specific toxicity, and allergic reactions, highlight the necessity of carefully assessing these medications in clinical settings. Ensuring patient safety and maintaining diagnostic accuracy necessitates a thorough understanding of the biocompatibility and potential risks associated with both Gd^3+^- and iron-based contrast agents [96]. As with any exogenous substance introduced into the body, MRI and CT CAs can lead to adverse effects. These may occur at multiple biological levels, including molecular, cellular, tissue, organ, and systemic, which necessitate careful preclinical and clinical evaluation [152–153]. Several factors influence the biocompatibility and safety of NCAs, including metal type and concentration, their electric and ionic charge, shape and surface of particles, and mechanism of clearance from the body [154]. Side effects differ between Gd-containing CAs and iron-containing CAs. The occurrence of these complications depends on various factors, notably the chemical composition of the contrast materials and the type of metal incorporated. Overall, most biocompatible NCAs demonstrate a lower incidence of allergic reactions and systemic toxicity compared to conventional CAs. Events such as hemolysis, coagulopathies, or disruptions in blood biochemical parameters have been reported only rarely with these agents. Therefore, considering both the necessity of using contrast in MRI imaging and the complications of current contrasts, biocompatible NCAs represent a suitable alternative in the future [155]. Collectively, these advancements suggest that biocompatible NCAs, including both Gd^3+^- and iron-based agents, offer safer, more effective alternatives for MRI and CT imaging, with the potential to enhance diagnostic performance and patient safety [108]. Advancing toward clinical translation will require scalable and reproducible manufacturing, careful evaluation of long-term biological interactions, and eventual human safety and imaging performance assessments [86]. These considerations outline the staged progression from nanoparticle design to clinical translation, which is summarized in the workflow shown in Figure 2.

### 5 Efficacy and diagnostic applications

The emergence of NCAs has significantly advanced the field of diagnostic imaging by enhancing the sensitivity, specificity, and resolution of MRI and CT scans. These nanoscale materials possess unique physicochemical properties that allow for improved visualization of anatomical structures and pathological changes, particularly in the early detection and staging of diseases such as cancer. Unlike traditional contrast agents, NCAs offer multifunctionality, including tumor targeting, enhanced signal intensity, and reduced systemic toxicity [3,145]. A summary of the major NCAs, their physiochemical characteristics, administration routes, and biocompatibility metrics is provided below in Table 4, and this section further outlines their efficacy in a range of oncological applications, highlighting their contributions to early diagnosis, targeted imaging, and improved clinical outcomes across various tumor types.

#### 5.1 Cancer detection and imaging

Cancer, characterized by uncontrolled cellular proliferation and metastatic potential, remains a critical global health challenge, with early detection being pivotal for successful treatment. Advanced imaging techniques such as MRI and CT scans, in combination with NCAs, have significantly enhanced the precision of tumor localization and characterization. These agents allow for high-contrast, high-resolution tumor imaging, contribute to improved diagnostic accuracy, and hold considerable promise for targeted imaging and therapeutic applications [1–2]. For instance, PEG-UCNPs-5 has demonstrated promise as a multimodal MRI/fluorescence probe for deep tissue imaging and early cancer detection [156]. Additionally, several NCAs function as positive MRI contrast agents with tumor-targeting capabilities, enabling visualization of tumor angiogenesis. In vivo *T*_1_-weighted MRI studies have proven that such agents can diffuse in solid tumors, leading to contrast enhancement at the periphery of a tumor and aiding in the differentiation between benign and malignant lesions [157].

**5.1.1 Breast cancer.** Breast cancer is one of the most actively investigated targets for NCA-based MRI, driven by the well-characterized overexpression of molecular markers enabling receptor-specific nanoparticle delivery. NCAs targeting CD44-overexpressing breast cancer cells using hyaluronic acid-modified MnFe_2_O_4_ nanocrystals demonstrated high sensitivity and specificity for the MRI detection of MDA-MB-231 and MCF-7 cells in vitro, while the optimized hyaluronic acid conjugation ratio promoted effective CD44 binding and maintained high cell viability [144]. These results establish a direct relationship between receptor expression level and imaging performance, supporting the potential of CD44-targeted NCAs for early detection of triple-negative and cancer-stem-cell-rich breast cancer subtypes. Beyond CD44, HER2 represents a second major molecular target for NCA-based breast cancer imaging. Dextran-coated SPIONs conjugated with Herceptin demonstrated MRI signal enhancement proportional to HER2/neu expression levels across multiple breast cancer cell lines in vitro, and produced a 45% signal drop at the tumor site in *T*_2_-weighted MRI following intravenous administration in mice bearing breast tumor allografts, confirming receptor-mediated targeting and in vivo accumulation [158]. Additionally, USPIO NCAs have demonstrated *T*_1_-weighted contrast enhancement at tumor peripheries consistent with active angiogenesis, supporting their use for visualizing tumor vascular architecture [157]. Together, these findings illustrate that ligand selection in breast cancer NCA design should be guided by tumor molecular subtype, CD44-targeted agents for triple-negative disease and HER2-targeted formulations for HER2-amplified subtypes, underscoring the need for biomarker-stratified NCA development.

**5.1.2 Brain tumors.** Gliomas, particularly glioblastoma, represent one of the most challenging targets for NCA-based imaging due to the selective permeability of the blood–brain barrier (BBB). Despite this, several NCAs have demonstrated meaningful brain tumor imaging performance in preclinical models. Transferrin-conjugated SPIONs (Tf-SPIONs) administered intravenously in C6 glioma-bearing rats produced significant *T*_2_-weighted contrast enhancement in tumor tissue that was still clearly visible at 48 h post-injection, attributed to receptor-mediated transcytosis of the transferrin-functionalized particles across the partially disrupted BBB and subsequent retention in tumor cells confirmed by Prussian blue staining [159]. Lactoferrin-conjugated magnetic poly(aminoethyl ethylene phosphate)–poly(ʟ-lactic acid) (PAEEP-PLLA) nanoparticles similarly produced strong, long-lasting glioma contrast enhancement in Wistar rats at 4 h post-injection, sustained over 48 h, pointing to receptor-mediated BBB crossing as the predominant uptake mechanism [160]. More recently, multimodal theranostic nanoparticles have been shown to combine MRI tumor tracking with concurrent therapeutic delivery in orthotopic glioblastoma models; ferumoxytol-FITC dual-mode nanoparticles enabled quantitative correlation between macroscopic *T*_2_ MRI contrast enhancement and microscopic intratumoral nanoparticle accumulation measured by intravital two-photon microscopy, providing one of the most rigorous in vivo validations of NCA tumor targeting in brain tumors reported to date [161]. Certain NCAs also exhibit potential for combined glioma imaging and photodynamic therapy [162].

**5.1.3 Renal cancer.** Renal cell carcinoma (RCC), which accounts for approximately 90% of renal malignancies, presents specific imaging opportunities given the overexpression of carbonic anhydrase IX (CAIX) on clear cell RCC cells. Carbonic anhydrase IX-targeted SPIO nanoprobes conjugated with monoclonal antibody G250, a CAIX-specific antibody, demonstrated selective in vitro labeling of clear cell RCC cells at 3.0 T MRI, producing significantly greater *T*_2_ signal attenuation in CAIX-positive cells compared to control cells, with no signal change in CAIX-negative lines [163]. Broader preclinical evidence supports the utility of NCAs for RCC visualization and staging, including favorable biodistribution profiles in vivo consistent with renal-clearable ultrasmall designs [164]. However, BBB penetration remains one of the most formidable translational challenges for NCAs in neurooncology. The BBB restricts the passage of most nanoparticles due to tight junctions between cerebral endothelial cells, efflux transporters, and size-dependent exclusion, meaning that passive accumulation via the EPR effect, reliable in peripheral tumors, is largely absent in intact brain parenchyma [165]. In glioblastoma, where the BBB is partially disrupted in the tumor core, some passive NCA accumulation has been reported, but peritumoral infiltrating cells, which carry the highest risk of recurrence, reside in regions where the barrier remains intact [166]. Strategies under active investigation to overcome this include receptor-mediated transcytosis via transferrin- or lactoferrin-functionalized nanoparticles, focused ultrasound-mediated transient BBB opening combined with NCA administration, and intrinsic BBB-permeable ultrasmall nanoparticle designs below 7 nm [165–166]. While promising in preclinical models, none of these strategies has yet achieved routine clinical validation for NCA-based brain tumor imaging, and this gap represents a priority area for future research.

**5.1.4 Liver cancer.** Liver cancer, and hepatocellular carcinoma (HCC) in particular, represents a high-priority target for NCA-based imaging given the critical importance of early detection for surgical eligibility and survival outcomes. MRI with conventional gadolinium-based agents and CT with iodinated agents remain the clinical standard for HCC diagnosis, but their limited sensitivity for lesions below 1 cm and inability to provide molecular information have motivated the development of nanoparticle-based alternatives [167–169]. NCAs have been investigated for HCC imaging across both *T*_2_-weighted MRI and CT modalities. Iron oxide-based NCAs exploiting Kupffer cell phagocytosis have been established as effective *T*_2_-weighted MRI agents for early-stage HCC and cirrhosis: Healthy hepatic parenchyma rich in functional Kupffer cells accumulates the particles and darkens on *T*_2_-weighted sequences, while HCC lesions deficient in Kupffer cells remain signal-bright, creating high-contrast lesion delineation. Extending this principle to active molecular targeting, ultrasmall paramagnetic iron oxide NCAs conjugated with anti-EGFR antibodies have demonstrated rapid and selective tumor uptake with efficient systemic clearance in preclinical HCC models, making them candidates for early-stage diagnosis where lesion size precludes detection by conventional agents [112]. For dual-mode ultrasound/MRI imaging of HCC, gadolinium-loaded phase-transition nanodroplets functionalized with folic acid have shown tumor-selective signal enhancement through folate receptor-mediated uptake, providing simultaneous ultrasound and MRI contrast in hepatic malignancy models [168]. Collectively, these approaches demonstrate that NCAs can address complementary diagnostic needs in HCC, passive Kupffer cell-based agents for parenchymal mapping, and actively targeted formulations for molecular lesion characterization, though clinical translation requires demonstration of consistent performance in the fibrotic and cirrhotic liver environments that characterize most HCC patients.

**5.1.5 Lung cancer.** Given the high mortality linked to late-stage detection, early lung cancer diagnosis is a critical priority. Anti-EGFR antibody-conjugated SPIONs administered intravenously in Lewis lung carcinoma (LLC1)-bearing C57BL/6 mice demonstrated selective tumor accumulation with good MRI signal intensity under both ex vivo and in vivo conditions, with significantly higher iron concentration at the tumor site compared to non-specific controls, confirming receptor-mediated targeting as the delivery mechanism [170]. Complementary work using intrapulmonary administration of surface-coated SPIONs confirmed that coating composition critically determines both biocompatibility and in vivo MRI detection efficiency in lung tissue, with PEG- and dextran-coated formulations showing superior performance compared to uncoated particles [35]. Together, these findings support the potential of NCA-based targeted MRI for early lung tumor detection, although translation requires demonstration of consistent tumor-to-background contrast at clinically relevant field strengths and in larger animal models.

In summary, NCAs represent a significant advancement in cancer imaging, offering enhanced specificity, improved biocompatibility, and targeted molecular interactions. Across multiple tumor types, including breast, brain, liver, and lung. These agents have demonstrated the potential to improve diagnostic accuracy, facilitate early detection, and support more precise therapeutic strategies. By consolidating the developments in design, functionality, and safety, NCAs exemplify a promising direction for the next generation of imaging agents in clinical practice.

#### 5.2 Non-oncological applications

Beyond oncology, NCAs hold considerable promise for imaging cardiovascular, neurological, and inflammatory diseases, areas where conventional agents frequently fall short due to their rapid redistribution and inability to accumulate at sites of molecular pathology. In cardiovascular imaging, USPIOs have been investigated as agents for detecting macrophage-rich atherosclerotic plaques, exploiting the phagocytic uptake of iron oxide particles by plaque-infiltrating macrophages to generate focal *T*_2_* signal loss in vulnerable plaque regions that are at highest risk of rupture, a capability inaccessible to conventional Gd-based agents, which distribute passively and provide no cellular specificity [171]. Nanoparticle probes targeting endothelial adhesion molecules, particularly VCAM-1 upregulated during early atherogenesis, have demonstrated the ability to detect vascular inflammation prior to the anatomical stenosis that triggers clinical symptoms, suggesting a pathway toward genuinely pre-symptomatic cardiovascular risk stratification [172]. Iron oxide nanoparticles functionalized with annexin-based ligands have additionally been used to image apoptotic cardiomyocytes in myocardial infarction models, providing spatial information about the extent of ischemic injury that conventional gadolinium enhancement cannot resolve at the cellular level [173]. Ferumoxytol, already available for clinical intravenous use, has been applied off-label for cardiovascular MRI including assessment of myocardial inflammation post-infarction and congenital heart disease vasculature in pediatric patients where gadolinium is contraindicated, underscoring the translational potential of iron-based NCAs in this domain [109].

In neurological imaging, iron oxide-based NCAs have demonstrated the ability to detect neuroinflammation, specifically macrophage and microglial infiltration, in multiple sclerosis lesions independently of BBB breakdown, a key limitation of Gd-DTPA which can only reveal active inflammation where barrier disruption has already occurred [174]. For Alzheimer’s disease, NCAs designed to exploit receptor-mediated transcytosis pathways and target amyloid-β plaques have shown MRI signal changes in preclinical models prior to the development of cognitive symptoms; however, confirmed BBB penetration in humans remains to be established, and the paradigm of presymptomatic neurodegeneration imaging through this approach is still in early preclinical stages [175]. These applications collectively illustrate that the molecular targeting capability of NCAs enables detection of pathological processes at stages where conventional agents are blind, shifting the role of contrast-enhanced MRI from anatomical delineation toward genuine molecular disease characterization. Extending rigorous preclinical validation and early-phase clinical studies into these non-oncological domains represents one of the most compelling opportunities for NCAs to demonstrate clinical value beyond their established oncological applications.

#### 5.3 Magnetic particle imaging and its NCA requirements

MPI is a tracer-based imaging modality first described by Gleich and Weizenecker in 2005; it detects the nonlinear magnetization response of SPIONs directly, without relying on tissue proton relaxation as in MRI [176]. Unlike MRI, MPI generates signal exclusively from the tracer itself, producing images with theoretically unlimited tissue contrast, no background signal from surrounding tissue, and no ionizing radiation, while offering high temporal resolution suitable for real-time vascular and cardiac imaging [176–177]. These properties position MPI as a potentially powerful complementary modality to MRI and CT, particularly for applications requiring dynamic blood-pool imaging, such as angiography, perfusion mapping, and tracking of cell-based therapies [177]. However, MPI remains in the preclinical stage, with no clinically approved tracers or scanners as of the time of writing. The central challenge is that MPI signal quality is exquisitely sensitive to nanoparticle properties in ways that differ substantially from MRI relaxivity requirements. Optimal MPI performance requires SPIONs with a narrow, monodisperse size distribution centered around 20–30 nm since particle-to-particle size heterogeneity broadens the magnetization transition that MPI detects and directly degrades spatial resolution and sensitivity [178]. Core crystallinity and the absence of surface spin disorder are equally important as magnetic moment uniformity across the particle ensemble determines signal sharpness. Surface coating must be thick enough to prevent interparticle dipolar coupling, which dampens the nonlinear response, while remaining thin enough to keep the hydrodynamic diameter within a range compatible with adequate circulation time and eventual clearance [178–179]. These requirements create design tensions not encountered in MRI-focused NCA development: SPIONs optimized for *T*_2_-weighted MRI contrast through large magnetic moment and strong field perturbation may produce broad, poorly resolved MPI signals if their size distribution is wide or their surface coating promotes aggregation. Conversely, the ultrasmall SPIONs favored for renal clearance and *T*_1_ MRI contrast fall well below the optimal size range for MPI [179]. Currently, Synomag^®^-D (micromod Partikeltechnologie) and LS-008 (LodeSpin Labs) represent the most extensively characterized research-grade MPI tracers, with LS-008 demonstrating substantially superior MPI performance to the clinically approved ferucarbotran (Resovist^®^), which was long used as a reference MPI tracer despite being designed for hepatic MRI rather than MPI [178]. Integrating MPI performance criteria into NCA design, alongside MRI relaxivity and CT attenuation, represents a meaningful opportunity to develop genuinely multimodal nanoparticle platforms, though the distinct and often competing physicochemical requirements of each modality make simultaneous optimization a non-trivial materials science challenge.

### 6 Comparative performance and emerging strategies

#### 6.1 Conventional CAs vs NCAs

Contrast agents enhance the diagnostic accuracy of MRI and CT imaging, particularly in detecting malignancies and vascular disorders [1–2]. While Gd-based *T*_1_ agents remain clinically prevalent, their rapid extravasation and risk of NSF have prompted the transition toward NCAs offering improved vascular retention, biocompatibility, and relaxivity [12,58,97]. Unlike conventional LMW chelates, NCAs display tunable core composition, size, and surface coatings that improve *r*_1_/r_2_ ratios and extend circulation time. These attributes enable high-resolution and multimodal imaging while reducing the limitations associated with traditional agents [1,6,12]. However, the comparative performance data summarized across Sections 6.2–6.5 must be interpreted with important caveats. Relaxivity values reported in the NCA literature are highly sensitive to measurement conditions, that is, field strength, temperature, solvent, and whether measurements are performed in buffer versus plasma; yet, these parameters are inconsistently reported and rarely matched to clinical field strengths of 1.5 or 3 T, where relaxivity for most agents differs substantially from values obtained at lower fields [180]. This makes headline *r*_1_ comparisons between investigational NCAs and approved agents such as Gd-DTPA (*r*_1_ = 3–5 mM^−1^·s^−1^ at 1.5 T) difficult to evaluate honestly since favorable NCA values are frequently measured under conditions that inflate performance relative to the clinical benchmark. Furthermore, the vast majority of efficacy data are derived from rodent models, where differences in vascular anatomy, immune surveillance, and metabolic clearance rates limit the predictive value for human pharmacokinetics [124]. Until relaxivity reporting is standardized and efficacy data are generated in larger animal models or early human studies with direct head-to-head comparisons against approved agents at equivalent doses, claims of superior diagnostic performance over existing clinical standards should be treated as preliminary.

#### 6.2 Dual-mode and multimodal imaging advances

NaGdF_4_:Yb^3+^ (50%) nanoparticles combine Gd and Yb for bimodal CT/MR imaging. Folic acid functionalization enhances uptake in folate-receptor-positive tumors, and a linear correlation between concentration and HU confirms CT efficiency. Their longitudinal relaxivity exceeds that of Gd-DTPA and the *r*_2_/*r*_1_ < 2 supports *T*_1_-weighted use [82]. PB@FePt-HA-*g*-PEG nanoparticles, integrating Prussian-blue and FePt components coated with PEG and hyaluronic acid, show CT enhancement and *T*_2_ signal attenuation, verifying dual-mode imaging. The hyaluronic acid shell provides tumor targeting, and in vivo studies demonstrate tumor growth inhibition [83]. FePt and Magnus nanobullets (Fe_3_O_4_@SiO_2_-Mn^2+^) yield signal-to-noise ratio (SNR) increases of 57–61% post-injection, demonstrating strong dual-mode performance [28,181–182]. Nanostructured coordination polymers (NCPs) containing Gd^3+^, Fe^3+^, or Mn^2+^ exhibit *r*_2_/*r*_1_ values between 0.6 and 2.1, allowing for combined *T*_1_/*T*_2_ imaging [183]. Together, these materials exemplify how NCAs bridge traditional single-modality imaging and next-generation precision theranostics.

#### 6.3 Polymeric and protein-based NCAs

Polymeric micelles based on *N*-(2-hydroxypropyl) methacrylamide (HPMA) containing Gd^3+^ displayed high longitudinal relaxivity (10.9 mM^−1^·s^−1^), approximately threefold higher than that of Gd-DTPA and enable superior MR angiography of tumors and vasculature in mice with longer imaging windows [12]. HFn-Gd, a ferritin-gadolinium complex combining Gd with human heavy chain ferritin (HFn), achieves *r*_1_ of 4.78 mM^−1^·s^−1^, versus 3.97 mM^−1^·s^−1^ for Gd-DTPA. Its larger molecular weight increased the rotational correlation time, enhancing relaxivity and reducing required Gd dosage. SNR values were consistently superior to Gd-DTPA, making HFn-Gd effective for early tumor detection [184]. Gd_2_O_3_@PCD-FA nanoparticles, coated with β-cyclodextrin-based polyester and functionalized with folic acid, showed targeted uptake in cancer cells. Compared to Gd-DOTA, they provided higher *T*_1_ relaxivity, better cytocompatibility, and improved imaging selectivity [19]. These results confirm that polymeric and protein scaffolds can enhance relaxivity while improving biocompatibility and targeting performance.

#### 6.4 Mn- and Fe-based dual-function agents

Ultrasmall manganese ferrite nanoparticles (Mn*_x_*Fe_3−_*_x_*O_4_, ≈3 nm) with Mn fractions up to *x* = 0.75 achieved maximal *r*_1_ relaxivity (10.35 mM^−1^·s^−1^). Further increases in Mn content reduced efficacy and increased toxicity. Mn_0.75_Fe_2.25_O formulation demonstrated strong liver contrast [185]. MNP-PEG-Mn, consisting of melanin nanoparticles chelated with Mn^2+^, exhibited an exceptional *r*_1_ of 20.57 mM^−1^·s^−1^, 3.4 times greater than that of gadodiamide. In vivo results showed strong tumor targeting and efficient renal and hepatobiliary clearance, minimizing accumulation-induced toxicity [13]. USPIOs stabilized with multidentate block copolymers (MDBCs) exhibited *r*_2_/*r*_1_ ratios of ≈4 and high *r*_1_ relaxivities (15.9–18.8 mM^−1^·s^−1^) with linear PEG blocks. These properties enhance vascular imaging and prolong circulation time [186]. These data highlight the potential of Fe/Mn hybrids for safe and efficient dual-mode MRI.

#### 6.5 Fluorescent and silica-coated hybrid nanoplatforms

Gd-FPNPs, Gd-chelate-conjugated polymers, demonstrated strong MR and fluorescence imaging capability. Their *r*_1_ values exceeded those of commercial agents, with a low *r*_2_/*r*_1_ ratio (≈1.06), and *T*_1_-weighted signal intensity improved by 37.1%, outperforming Gadobutrol [187]. Fe@FeOx@SiO_2_ nanoparticles with 11 nm silica shells show high water stability, no cytotoxicity after 72 h, and no hemolytic activity, making them suitable for *T*_2_ imaging [188]. Naumenko et al. directly compared magnetic nanocubes (MNCb), nanoclusters (MNCl), and nanorods (MNRd) in multiple murine tumor models, providing one of the most systematic experimental demonstrations of morphology-dependent NCA performance in vivo. MNCb showed the highest *r*_2_ relaxivity and tumor delivery efficiency, consistent with their flat facets promoting closer membrane contact and efficient endocytosis as discussed in Section 2. MNCl yielded the greatest tumor contrast enhancement at 96%, followed by MNCb at 73% and MNRd at 63%, a ranking that reflects the interplay between cluster-induced collective magnetic behavior, shape-dependent vascular margination, and differential mononuclear phagocyte system recognition across morphologies [30]. These findings reinforce that morphology is an independently controllable determinant of in vivo imaging performance that cannot be predicted from composition or size alone, and that systematic shape screening, as conducted in this study, should become a standard component of NCA preclinical development pipelines.

#### 6.6 Summary

NCAs surpass conventional contrast agents through enhanced relaxivity, improved vascular retention, and the potential for multimodal or theranostic applications [180]. Composition-specific innovations, including polymeric, protein, Mn/Fe-based, and silica-fluorescent hybrids, are guiding MRI toward higher precision and clinical translatability. Continued evaluation of long-term safety and biodegradation will be crucial for regulatory integration.

**Table 2:** Pharmacokinetics, stability, and side-effect profiles by NCA material class.

Material class	Representative agents	Stability/clearance pathway	Reported side effects	Size dependence

Gd-based [19,32,72,98–103]	Gd-MCNs, FS-GdNDs, Gd-CHPOA, Gd_2_O_3_@PCD-FA, GTNs	predominantly hepatobiliary clearance (Gd-MCNs); long-term stability confirmed up to three months (Gd-CHPOA, no histopathological abnormalities)	low ROS generation, minimal cytokine production, hemolysis below 3–6%; uncoated Gd_2_O_3_ shows notable cytotoxicity from Gd^3+^ leakage, mitigated by coating	coating composition (PCD, CHPOA) more decisive than core size for cytotoxicity in reported studies
Fe-containing [28,36,39,93,104–108,110–123]	ZUIONs, Fe_2_O_3_/Fe_3_O_4_, Fe_2_O_3_@BSA, SPIONDex	renal clearance (ZUIONs); structurally stable in vivo; efficient metabolic clearance broadly reported	minimal hemolysis, no detectable organ injury; SPIONDex avoids complement activation and CARPA seen with clinical agents (ferumoxytol, ferucarbotran)	hydrodynamic size (alongside surface charge/coating) determines protein corona formation, MPS recognition, and renal vs hepatosplenic clearance balance
Mn-based [52,54,56,94,125–127]	MnSNs, MnO_2_/PAA	rapid clearance: major organs within 24 h, kidneys within 72 h; minimal tissue retention	low systemic toxicity, no histological abnormalities; toxicity modulated by surface coating limiting ion leakage under acidic endosomal conditions	particle size, surface coating, and charge collectively influence uptake, ion release, and oxidative stress
C-based [60,128]	A-C-dots@Ce6, CDs@PEI	CDs <6 nm: predominantly renal excretion; CDs >8 nm: hepatic processing and potential bioaccumulation; hepatobiliary excretion confirmed in vivo for multi-CD nanoparticles (CDs@PEI)	nontoxic at high concentrations (A-C-dots@Ce6, a Gd^3+^-assembled theranostic system providing *T*_1_ MRI contrast via incorporated Gd^3+^ ions); toxicity of C-dots broadly dependent on synthesis method, surface charge, and chemical composition	strong size dependence: <6 nm → renal clearance, low toxicity; >8 nm → hepatic route, higher bioaccumulation risk; surface functionalization modulates all clearance parameters
lanthanide-doped (UCNCs) [47–51]	NaYF_4_:Yb,Tm (PEO-PAA coated)	retained in cardiac tissue up to 15 days post-injection (intramyocardial/intrapericardial); no detectable accumulation in liver/kidney from cardiac sites	no alteration of heart rate, heart rate variability, or left ventricular ejection fraction over 15 days; minimal to no cardiac tissue necrosis (pericardial site)	≈38 nm hexagonal particles evaded lymphatic clearance from the pericardial space despite exceeding typical pericardial fenestration size
quantum dots [63]	CdSe, CdTe, InP, ZnS-capped	size-dependent: <6 nm → renal clearance; larger QDs → hepatic processing and potential accumulation; incomplete clearance documented for heavy-metal formulations	heavy-metal-based (Cd, Te): dose-dependent cytotoxicity, metal ion leaching; heavy-metal-free (InP, carbon-based): improved safety but generally lower imaging performance	strong size dependence of clearance route; surface coating critically modulates metal ion leaching and toxicity

**Table 3:** Clinically approved conventional contrast agents for MRI and CT serving as benchmarks for NCA development.

Agent (trade name)	Modality	Composition	Primary clinical use	Key limitations

gadolinium diethylenetriamine pentaacetic acid (Magnevist^®^) [58,96]	MRI (*T*_1_)	linear ionic Gd^3+^-DTPA chelate	CNS, spinal, and vascular imaging	NSF in renal impairment; gadolinium deposition in brain and bone with repeated use
gadobutrol (Gadovist^®^) [58,97]	MRI (*T*_1_)	macrocyclic Gd^3+^-DOTA-based chelate	CNS and vascular imaging	lower NSF risk than linear agents; gadolinium deposition still reported
iohexol (Omnipaque^®^) [20–21]	CT	low-osmolar nonionic iodinated monomer	CT angiography, body and urographic CT	contrast-induced nephropathy in at-risk patients; hypersensitivity reactions
ferumoxides (Feridex^®^/Endorem^®^) [36,108]	MRI (*T*_2_)	dextran-coated iron oxide; 120–180 nm	hepatic MRI (liver tumor detection)	withdrawn; acute hypersensitivity, manufacturing instability, suboptimal relaxivity
ferucarbotran (Resovist^®^) [36,108,120]	MRI (*T*_2_/*T*_1_)	carboxydextran-coated iron oxide; ≈60 nm	hepatic MRI; bolus-injectable	withdrawn; complement activation, CARPA, cytotoxicity
ferumoxytol (Feraheme^®^) [109]	MRI (*T*_1_/*T*_2_)	polyglucose sorbitol carboxymethylether-coated SPIO; ≈30 nm	iron deficiency anemia; off-label blood-pool MRI agent	FDA boxed warning; serious hypersensitivity reactions

**Table 4:** Summary of NCAs reported in MRI and CT imaging, including their physiochemical properties, administration, and biocompatibility.

NCA	Study type	Imaging modality	Properties	Administration/circulation	Biocompatibility metrics

C-dots [55,59]	in vitro, in vivo	fluorescence/MRI/CT (doped forms)	<5 nm (TEM, mean physical diameter); high water solubility; surface-modifiable	short blood circulation	non-toxic
SPIONs [17]	in vivo (Fe_3_O_4_, γ-Fe_2_O_3_)	MRI	few nanometers to >100 nm (hydrodynamic diameter by DLS, population range across formulations); water-dispersible; variable surface coatings	variable	good biocompatibility; generally superior to GBCAs
UCNCs [47,51]	in vivo (cardiac model); in vitro synthesis and multimodal characterization	UCL/fluorescence imaging; UCL/CT/MRI demonstrated for NaGdF_4_:Yb,Ln class	≈38 nm (TEM, mean physical diameter); NaGdF_4_:Yb,Ln core; water-dispersible; PEO-PAA polymer coating	intramyocardial/intrapericardial	no cardiac functional impairment over 15 days; minimal necrosis at pericardial site
Gd-MCNs [32]	in vivo, in vitro	MRI	mesoporous carbon nanoparticles with high relaxivity (39.044 mM^−1^·s^−1^ Gd)	intravenous	high biocompatibility; lower inflammation/toxicity vs Gd-DTPA
Gd-CHPOA [72]	in vivo	MRI	assembled from cholesterol-modified and acryloyl-modified pullulan with Gd^3+^; water-dispersible; hydrodynamic size ≈65 nm	intravenous	no significant cytotoxic effects; no histopathological abnormalities up to three months
Gd^3+^-doped titania [103]	in vitro, clinical trial	MRI	25 nm (TEM, mean physical diameter); water-dispersible; undoped TiO_2_ and Gd^3+^-doped variants	intravenous	no significant cytotoxicity; minimal inflammatory response; normal coagulation parameters
SPIONDex [108]	in vitro, in vivo	MRI	hydrodynamic size ~30–130 nm (variant-dependent); water-dispersible; cross-linked dextran coating	intravenous	no cytotoxicity in human macrophages, endothelial cells, or monocytes; no complement activation or CARPA
Fe_2_O_3_@BSA [39]	in vitro, in vivo	MRI	6–7 nm (TEM, mean physical diameter); water-dispersible; BSA protein shell	intravenous	minimal cytotoxicity up to 300 µg·mL^−1^ Fe; no organ abnormalities at 7 and 14 days post-injection
C-dots@Ce6 [128]	in vitro, in vivo	MRI (*T*_1_)/fluorescence/PDT	size not reported; water-dispersible; Gd^3+^ ions as electrostatic cross-linker and *T*_1_ contrast source; Ce6-photosensitizer-loaded	intravenous *(*implied/standard for theranostic delivery*)*	negligible cytotoxicity at high concentrations
Gd/Ru-doped C-dots [61]	in vitro, in vivo	MRI/fluorescence	metal-ion-doped carbon matrix; not reported (size); water-dispersible	intravenous	low cytotoxicity; suitable biocompatibility confirmed in vitro and in vivo
Gd/Yb co-doped C-dots [62]	in vitro, in vivo	MRI/CT/fluorescence	≈5.26 nm (TEM, mean physical diameter); hydrothermal synthesis; Gd/Yb dual-doped; water-dispersible	intravenous	renal clearance confirmed; no significant organ toxicity
CZTS-BSA [141]	in vivo	MRI/CT	size not reported; water-dispersible; BSA conjugation	intravenous	low cytotoxicity; no hepatotoxicity, nephrotoxicity, or hematological abnormalities
DOTA-modified CNCs [140]	in vitro	MRI/CT	rod-shaped; 5–20 nm width, 100–500 nm length (TEM, physical dimensions); water-dispersible; DOTA surface functionalization	intravenous/ suspension (in vitro test)	low cytotoxicity
RBC membrane-camouflaged nanoparticles [189]	in vivo	MRI	size not reported; water-dispersible; RBC membrane coating	intravenous	no significant cytotoxicity
PEG-modified gadolinium nanoparticles [131]	in vitro, validated in vivo	CT	size not reported; water-dispersible; PEG coating	intravenous	no significant cytotoxicity
NaGdF_4_:Yb^3+^ (50%) [82]	in vitro, in vivo	MRI/CT	ultrasmall (TEM); water-soluble; folic acid surface functionalization	intravenous	favorable biocompatibility profile in vivo (confirmed via animal monitoring)
PB@FePt-HA-g-PEG [83]	in vivo	MRI/CT/ PI	size not reported; Prussian blue and FePt components; HA-grafted PEG coating	intravenous	favorable biosafety in vivo
superparamagnetic contrast from FePt [28]	in vitro (MRI characterization)	MRI (*T*_1_/*T*_2_)	core: 2–8 nm (TEM, mean physical diameter); hydrodynamic diameter: 7.2–14.2 nm (DLS, amphiphilic polymer coating	not reported (in vitro study)	no apparent cytotoxicity at high concentrations; polymer coating limits Pt ion release
Magnus nanobullets [182]	in vivo	MRI	assembled from Fe_3_O_4_ nanoparticles with SiO_2_ shell and Mn^2+^; water-dispersible; not reported (size)	intravenous	good biocompatibility in vivo
NCPs (Gd, Fe, Mn) [183]	in vitro, in vivo	MRI (*T*_1_/*T*_2_)	size not reported; water-dispersible; coordination polymer matrix	intravenous	low cytotoxicity
HPMA combined with Gd^3+^ [12]	in vivo	MRI	size not reported; water-dispersible; HPMA polymer-Gd^3+^ micelle	intravenous	excellent biosafety
HFn-Gd [184]	in vivo	MRI	size not reported; human ferritin heavy chain shell with Gd^3+^	intravenous	high biosafety and biocompatibility in vivo
Gd_2_O_3_@PCD-FA [19]	in vitro	MRI	size not reported; β-cyclodextrin-based polyester (PCD) shell; folic acid targeting ligand	targeted cellular incubation (in vitro)	lower cytotoxicity vs uncoated Gd_2_O_3_; hemolysis < 6%
Mn*_x_*Fe_3−_*_x_*O_4_ [185]	in vitro, in vivo	MRI	≈3 nm (TEM, mean physical diameter); water-dispersible	intravenous	higher Mn content associated with higher cytotoxicity
MNP-PEG-Mn [13]	in vivo	MRI	size not reported; water-soluble; PEG coating; melanin nanoparticle core chelated with Mn^2+^	intravenous	favorable in vivo biosafety
MDBC/PEG [186]	in vitro (colloidal stability), in vivo (imaging performance)	MRI	size not reported; multidentate block copolymer with linear PEG blocks	intravenous	safe and biocompatible in vivo
Gd-FPNPs [187]	in vitro (biocompatibility metrics), potential in vivo (imaging performance)	MRI/fluorescence	size not reported; Gd-chelate-conjugated fluorescent polymer	intravenous	no cytotoxic effect, favorable immune response
Fe@FeOx@SiO_2_ [188]	in vitro	MRI	11 nm silica shell (TEM, physical shell thickness); water-stable; silica (SiO_2_) coating	aqueous suspension (in vitro)	no cytotoxicity; no hemolytic activity
MNCb (IONPs) [30]	in vitro, in vivo	MRI	Pluronic F-127-coated; water-dispersible; magnetic nanocubes	intravenous	safe and biocompatible; *r*_2_ = 300 mM^−1^·s^−1^ (in vitro); tumor contrast enhancement in 73% of tumors
MNCl (IONPs) [30]	in vitro, in vivo	MRI	Pluronic F-127-coated; magnetic nanoclusters	intravenous	safe and biocompatible; *r*_2_ = 104 mM^−1^·s^−1^ (in vitro); tumor contrast enhancement in 96% of tumors
MNRd (IONPs) [30]	in vitro, in vivo	MRI	Pluronic F-127-coated; magnetic nanorods	intravenous	safe and biocompatible; *r*_2_ = 51 mM^−1^·s^−1^ (in vitro); tumor contrast enhancement in 63% of tumors

## Conclusion

In conclusion, this review highlights the growing importance of biocompatible NCAs in MRI and CT imaging. These agents provide safer, more effective alternatives to conventional contrast agents by improving image resolution, reducing toxicity, and enhancing diagnostic precision. Evidence from preclinical and early clinical studies indicates that NCAs can significantly expand diagnostic capabilities while maintaining favorable safety profiles. Moreover, advances in multimodal and tumor-targeted formulations illustrate their potential for dual diagnostic and therapeutic applications. However, standardized clinical trials and long-term safety assessments are still required to validate their clinical translation. Collectively, these findings underscore the transformative role of NCAs in shaping safer, more personalized, and more effective diagnostic imaging strategies for the future.

## Future Priorities

To accelerate clinical adoption, future studies should focus on unified preclinical safety pipelines, comprehensive long-term biodistribution and clearance analyses in relevant animal models, and scalable good-manufacturing-practice production routes. Early-phase trials that correlate imaging improvements with clinical outcomes will also be essential. Establishing community-wide standards, similar to those emerging for other nanomedicines, would reduce duplication of effort and streamline translation [3,46,86]. Among the NCA categories summarized in Figure 1, hybrid and targeted constructs represent the most promising candidates for clinical translation owing to their multimodal and multifunctional design flexibility. The emergence of magnetic particle imaging as a preclinical modality offers an additional translational direction, and designing SPIONs that simultaneously satisfy MPI performance criteria and MRI or CT contrast requirements represents a compelling materials challenge for next-generation NCA development [176,179].

## Data Availability

Data sharing is not applicable as no new data was generated or analyzed in this study.
